# Efficacy and safety of traditional Chinese medicine in the treatment of menopause-like syndrome for breast cancer survivors: a systematic review and meta-analysis

**DOI:** 10.1186/s12885-023-11789-z

**Published:** 2024-01-08

**Authors:** Runxi Wang, Yan Wang, Liyuan Fang, Yi Xie, Shuhan Yang, Suying Liu, Yuhang Fang, Ying Zhang

**Affiliations:** 1 Department of Oncology, Guang’anmen Hospital, China Academy of Chinese Medical Sciences, Beijing, China; 2 Beijing University of Chinese Medicine, Beijing, China

**Keywords:** Breast cancer, Endocrine therapy, Menopause-like syndrome, Systematic review, meta-analysis

## Abstract

**Background:**

In recent years, breast cancer (BC) incidence and mortality have been the highest in females. Menopause-like syndrome (MLS), arising from hypoestrogenism caused by endocrine therapy, significantly affects the quality of life for females. Traditional Chinese Medicine (TCM) has advantages in ameliorating MLS, but the efficacy of TCM in patients with BC has not been systematically evaluated.

**Methods:**

A comprehensive search was performed on PubMed, Web of Science, Embase, Ovid, Cochrane Library, China National Knowledge Infrastructure, Wanfang database, Chinese Scientific Journals Database, and Clinical Trial Registry from inception to September 4, 2023. The Cochrane Risk of Bias assessment tool was used for the quality evaluation of the randomized controlled trials (RCTs). Review Manager 5.4 software was used for statistical analysis, and the Grading of Recommendations Assessment, Development, and Evaluation was used for quality evaluation of the synthesized evidence.

**Results:**

This review included 42 studies involving 3112 female patients with BC. The results showed that the TCM group was better at decreasing the Kupperman Menopausal Index (KMI) scores (standardized MD, SMD = − 1.84, 95% confidence interval, CI [− 2.21–-1.46], Z = 9.63, *P* < 0.00001). Regarding the main symptoms of MLS, the TCM groups could significantly decrease the scores of hot flashes and night sweats (SMD = − 0.68, 95% CI [− 1.1–-0.27], Z = 3.24, *P* = 0.001), paraesthesia (SMD = − 0.48, 95% CI [− 0.74–-0.21], Z = 3.53, *P* = 0.0004), osteoarthralgia (SMD = − 0.41, 95% CI [− 0.6–0.21], Z = 4.09, *P* < 0.0001), anxiety (MD = − 0.85, 95% CI [− 1.13, − 0.58], Z = 6.08, *P* < 0.00001) and insomnia (MD = − 0.61, 95% CI [− 0.8, − 0.43], Z = 6.51, *P* < 0.00001). TCM can effectively improve the symptoms of MLS in patients with BC. Moreover, TCM could improve the objective response rate (ORR) by 50% (RR = 1.5, 95% CI [1.37–1.64], Z = 9.01, *P* < 0.00001). Follicle-stimulating hormone (FSH) and oestradiol (E_2_) had no significant difference compared with the control group (*p* = 0.81 and *p* = 0.87), and luteinizing hormone (LH) in the TCM group decreased significantly (MD = − 0.99, 95% CI [− 1.38, − 0.5], Z = 5.01, *P* < 0.00001). This means that the use of TCM does not negatively affect endocrine therapy and may even have a synergistic effect. The incidence of adverse events (AEs) was lower in the TCM groups than in the control groups.

**Conclusions:**

The meta-analysis stated that TCM could better improve the MLS of patients, alleviate related symptoms, and did not increase adverse drug reactions in BC survivors. This review brings more attention to MLS, and the present findings shed light on the potential applications of TCM in the treatment of MLS in BC survivors.

**Supplementary Information:**

The online version contains supplementary material available at 10.1186/s12885-023-11789-z.

## Introduction

Breast cancer (BC) describes a range of malignancies occurring in the mammary glands and is the most prevalent cancer worldwide [1]. According to the latest statistics, there were 2,261,419 new cases of BC and 684,996 BC-related deaths in 2020, accounting for the first and sixth highest number of diagnosed cancer cases and deaths, respectively, worldwide [2]. The National Institutes of Health (NIH) estimated that the number of new BC cases in 2024 would be 287,850, accounting for 15% of all new cancer cases. Meanwhile, 43,250 new deaths will occur in 2024, accounting for 7.1% of all cancer deaths [3]. To prolong the life of patients and improve survivors’ quality of life, it is increasingly important to improve and enrich BC treatment [4]. Among BC treatments, endocrine therapy refers to the systematic use of aromatase inhibitor (AI) or oestrogen receptor (ER) modulators to reduce female hormone levels, inhibit ovarian function, and control tumour growth in female BC survivors who are ER-positive [5]. Notably, the ovarian damage caused by endocrine therapy could significantly reduce the secretion of oestrogen, which could significantly influence the function of the female endocrine system [6]. Due to endocrine therapy, female hormones suddenly and unnaturally drops, resulting in symptoms similar to menopause, such as hot flashes and night sweats, paraesthesia, osteoarthralgia, anxiety, insomnia, etc., which is called menopause-like syndrome (MLS), seriously affecting the patients’ quality of life [7]. Female hormones are an important indicator in the process of endocrine therapy, and inhibiting the increase in female hormones is of great significance in controlling the development of BC [8]. How to relieve MLS and improve the quality of life of patients without increasing female sex hormones is very important.

Traditional Chinese Medicine (TCM) is an important source of antitumour drugs. Approximately 50% of the currently used antitumour drugs are directly or indirectly derived from TCM, including various compounds, such as alkaloids, polysaccharides, polyphenols, diterpenes, and unsaturated fatty acids [9, 10]. TCM can reduce the development of tumour resistance and inhibit malignant metastasis of tumour cells [11]. Moreover, in addition to their effects on cancer cells, some TCMs exhibit other biological activities in noncancer cells, such as antioxidant, anti-inflammatory, and immunomodulatory activities, thereby enhancing patient immunity, increasing therapeutic efficacy, and reducing toxicity [12, 13].

Several clinical trials have explored the efficacy of TCM in MLS in BC survivors. A trial found that ribociclib plus endocrine therapy improves the progression-free survival (PFS) period and has controllable safety in premenopausal, hormone receptor (HR)-positive and human epidermal growth factor receptor-2 (HER 2)-negative BC patients compared with the control group [14]. Another multicentre, open-label, randomized, controlled, phase 3 trial suggested that the combination of TCM and endocrine therapy may be a potential treatment option for ER-positive and HER 2-negative primary BC patients at high risk [15]. However, no systematic review has been found in evaluating this topic based on recent evidence. Therefore, this review aimed to systematically assess the efficacy and safety of TCM in the treatment of menopause-like syndrome (MLS) in BC survivors using recently available evidence.

## Materials and methods

### Registration

We registered this systematic review and meta-analysis with PROSPERO (CRD42022316111). This study was conducted in accordance with the Preferred Reporting Items for Systematic Reviews and Meta-Analyses (PRISMA) guidelines [16]. The PRISMA checklist is available in Supplementary file 1. Since this study was based on data extracted from published trials, ethical approval was not needed.

### Eligibility criteria

#### Patients

Patients with a definite pathological diagnosis of BC with oestrogen receptor (ER)-positive patients and without any other malignancies was included in this study. Patients with MLS who have received or are currently undergoing endocrine therapy. There was no restriction in terms of cancer stages and menstrual cycles.

#### Intervention

Chinese herbal medicine was a requirement for patients in the experimental groups. There were no restrictions on Chinese herbal medicine forms, therapeutic dose, or frequency of administration. TCM and other interventions can exist in tandem.

#### Comparison

Placebo or blank controls were included.

#### Outcomes

The primary outcomes included the MLS status, which was evaluated by menopause assessment scales (Kupperman Menopausal Index [KMI] [17]. Secondary outcomes were female hormones, including E_2_, FSH and LH. Objective Response Rate (ORR) as evaluated by the Response Evaluation Criteria in Solid Tumours [18]. The safety outcome indicators were adverse events (AEs).

#### Study design

Randomized controlled trials (RCTs) with or without blinded methods were included in this study.

### Exclusion criteria

(1) Data extracted from the articles were insufficient, or the full text could not be obtained; (2) duplicate publications in different databases; (3) outcome indicators did not meet the requirements of this study.

### Search strategy

A comprehensive search included searching for PubMed, Web of Science, Embase, Ovid, Cochrane Library, China National Knowledge Infrastructure, Wanfang database, Chinese Scientific Journals Database, and Clinical Trial Registry from the inception of each until September 4, 2023. The MeSH terms and free terms were used to conduct the search. The search steps are supplied in Supplementary file 2.

### Study selection

All search results were imported into EndNote X9 (Clarivate, London, United Kingdom). Two independent researchers conducted title, abstract, and full-text screening of the included trials. All disagreements were resolved by discussion or by the intervention of a third researcher when needed. The SD of the change from baseline for the experimental intervention was input using the following formula [19]:

$${\textrm{SD}}_{\textrm{E},\textrm{change}}=\surd \left[{{\textrm{SD}}^2}_{\textrm{E},\textrm{baseline}}+{{\textrm{SD}}^2}_{\textrm{E},\textrm{final}}-\left(2\times \textrm{Corr}\times {\textrm{SD}}_{\textrm{E},\textrm{baseline}}\times {\textrm{SD}}_{\textrm{E},\textrm{final}}\right)\right];\textrm{Corr}=0.75.$$

The mean value of the change from baseline for the experimental intervention was input using [20]:

$${\textrm{Mean}}_{\textrm{E},\textrm{change}}={\textrm{Mean}}_{\textrm{E},\textrm{final}}-{\textrm{Mean}}_{\textrm{E},\textrm{baseline}}$$

all data were rounded to two decimal places.

### Data extraction

Data extracted from the RCTs included names of authors, year of publication, number of patients, method of randomization, mean age, type of disease, duration of treatment and outcomes. The original authors were contacted by email when necessary to obtain any missing data or to inquire about errors or ambiguous information.

### Quality assessment

Version 2 of the Cochrane Risk of Bias Tool (RoB 2) of the included RCTs was evaluated as low risk, some concerns, and unclear risk based on the evaluation of the following domains: selection bias, performance bias, detection bias, attrition bias, reporting bias, and other biases [14].

### Statistical analysis

The meta-analysis was conducted using Review Manager version 5.4 (Cochrane Collaboration, 2020). Random-effects and fixed-effects models were selected for the meta-analysis. When I2 > 50, the random-effects model was chosen. Otherwise, the fix-effects model was selected. Sensitivity or subgroup analyses were conducted to determine the cause of heterogeneity if it existed. The risk ratio (RR) was used to evaluate dichotomous outcomes, while the mean difference (MD) or standardized MD (SMD) was used to assess continuous variables. The MD was selected when these variables were all obtained using the same rating instrument, and the SMD was selected when different scales were used to measure the same outcome [21]. The effect estimates with their 95% CIs are reported herein. The I^2^ inconsistency index was used to quantify heterogeneity. Funnel plots were used to verify bias when there were more than 10 studies. The results of the sensitivity analysis are also reported herein.

### Quality of the synthesized evidence

Quality assessment of the synthesized evidence was performed using the Grading of Recommendations Assessment, Development, and Evaluation (GRADE) approach. Evidence quality could be downgraded by five factors: RoB, heterogeneity, indirectness, imprecision, and publication bias. The quality of evidence was rated as high, moderate, low, or very low.

## Results

### Literature search

**Fig. 1:**
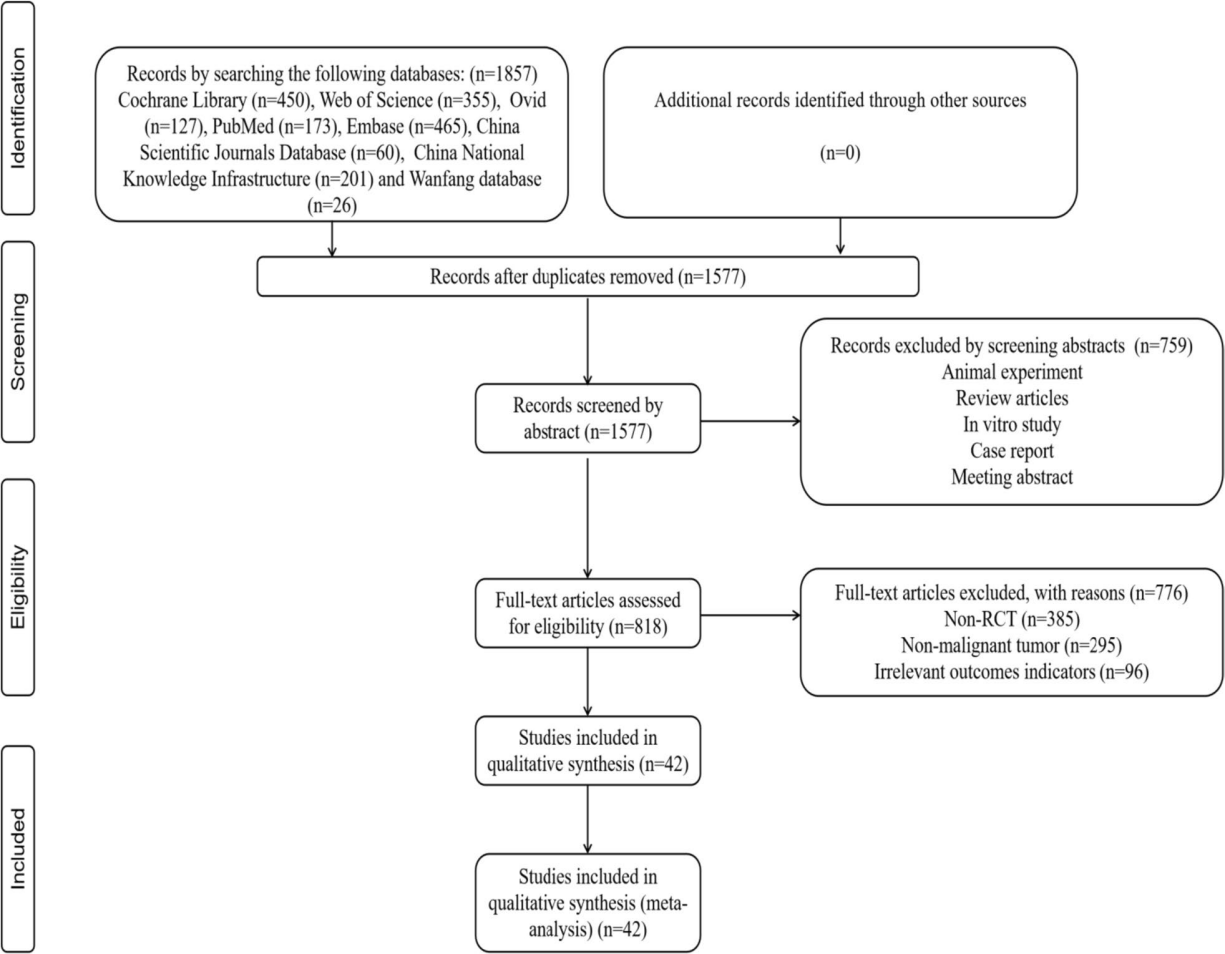
Literature screening process

According to the search strategy, a total of 1577 articles were retrieved. After reading the title and abstract, 759 articles were excluded. After screening these articles according to the search strategy and exclusion criteria, 385 articles were eliminated as follows: there were no RCTs, 295 did not meet the inclusion criteria, and 96 studies lacked outcome measures. Forty-two articles (Shi, 2010; Liang et al., 2018; Nie, 2018; Sun, 2009; Wang et al.,2019; Van Patten et al.,2002; Gao, 2019; Li et al., 2021; Tan, 2014; Lu et al., 2016; Xiao et al., 2019; Li et al., 2020; Yang et al., 2015; Yang et al., 2016; Liu et al., 2016; Zhang, 2018; Song, 2019; Zhou, 2020; Jacobson et al.,2001; Pei et al., 2019; Cai et al., 2021; Xu, 2019; Luo et al., 2019; Zhu, 2020; Sheng, 2014; Ou, 2018; Feng et al.,2021; Jiang, 2021; Qiang, 2020; Sui, 2019; Lin et al., 2016; Li et al., 2020; Zhu,2020; Wang, 2018; Wu et al., 2021; Liang, 2011; Fu, 2016; Li et al., 2022; Song et al., 2014; Tao et al., 2020; Han et al., 2021; Yan, 2021) were further analysed (Fig. 1). Table 1 reports the characteristics of the included studies. The composition and dosage of TCM included in the article are shown in Supplementary file 3.

**Table 1:** Characteristics of reports extracted from the databases

Study	Randomized method	Sample size (E/C)	BC’s stage	Menstrual cycle	Age	Experimental group	Control group	Duration	Outcomes
Bailing Shi, 2010;China	Random number table method	12/13	N	Post-menopausal	EG: 60.00 ± 4.84 CG: 61.78 ± 3.3	Traditional Chinese Medicine (TCM) + Aromatase inhibitor(AI) + Calcium tablet	AI+Calcium tablet	24 w	⑧
Chenlu Liang et al., 2018; China	Random number table method	42/43	I II III	Premenopausal	EG: 37.74 ± 6.6 CG: 37.16 ± 6.28	Black Cohosh+Luteinizing hormone releasing hormone analogue-α(LHRH-α)	LHRH-α	12 w	⑧⑨
Chen Nie, 2018;China	Not reported	20/20	I II	Premenopausal	EG: 41.1 ± 4.3 CG: 43.65 ± 5.36	Yiqiyangyinjiedu(YQYYJD) formula+Tamoxifen	Tamoxifen	12 w	①④⑤⑥
Chen Sun, 2009;China	Not reported	30/30	N	N	Not reported	Xiaoyaoerxian(XYEX) Decoction+Tamoxifen	Tamoxifen+Oryzanol	12 w	①⑦⑧⑨
Chen Wang et al.,2019;China	Randomized envelop method	42/43	I II III	Premenopausal	EG: 37.74 ± 6.60 CG: 37.16 ± 6.28	Black Cohosh+LHRH-α	LHRH-α	12 w	⑧⑨⑩
Cheri L. Van Patten et al.,2002; American	Not reported	59/64	I II III	Post-menopausal	EG: 55.5 ± 6.3 CG: 54.9 ± 6.5	Soy	placebo	12 w	⑪
Chundi Gao, 2019; China	Random number table method	24/24	N	N	EG: 44.13 ± 4.875 CG: 45.25 ± 4.386	Erxian(EX) Decoction+Tamoxifen	Tamoxifen	12 w	①⑦⑧⑨⑩
Congshan Li et al., 2021;China	Random number table method	30/30	I II III	Premenopausal	EG: 44.18 ± 3.39 CG: 44.97 ± 3.17	Chaihujialonggumuli(CHJLGML) Decoction+Oryzanol	Oryzanol	12 w	①②③④⑤⑥⑦
Guilan Tan, 2014;China	simple randomization	16/16	I II III	Post-menopausal	EG: 60.36 ± 8.64 CG: 59.00 ± 4.76	Bushen(BS) formula+AI+Calcium tablet	AI+Calcium tablet	Not reported	①⑧
Haisong Lu et al., 2016;China	Random number table method	30/30	N	Premenopausal	EG: 35 ~ 50 CG: 34 ~ 50	Xiaogeng(XG) powder+Tamoxifen	Tamoxifen+Oryzanol+Vitamin B6	8 w	⑧⑨⑩
Han Xiao et al., 2019;China	Random number table method	25/25	EG:I1 5 II 11 CG:I1 7 II 10	Post-menopausal	EG: 60.8 ± 8.7 CG: 62.1 ± 9.4	Jiaweizhibaidihuang(JWZBDH) Decoction+AI+Oryzanol	AI+Oryzanol	8 w	①⑧⑨
Hongxia Li et al., 2020; China	Random number table method	47/49	EG:I1 1 II 28 III 8 CG:I1 2 II 30 III 7	Premenopausal	EG: 42.3 ± 6.5 CG: 42.5 ± 7.1	Qianyangfengsui(QYFS) pellet+Oryzanol	Oryzanol	12 w	①⑦⑧
Huifen Yang et al., 2015;China	Not reported	69/33	N	Premenopausal	Not reported	Erzhi(EZ) pill +Guizhi(GZ) Decoction	Oryzanol	12 w	⑧⑨⑩
Huifen Yang et al., 2016;China	Random number table method	69/69	EG:I 22 II 38 III 9 CG:I 19 II 39 III 11	Premenopausal	Not reported	Erzhi(EZ) pill +Guizhi(GZ) Decoction	Oryzanol	8 w	①⑧⑨⑩
Hui Liu et al., 2016;China	Not reported	34/34	EG:II 25 III 10 CG:II 33 III 12	Premenopausal	Not reported	Yiguan(YG) Decoction+LHRH-α + AI	LHRH-α + AI	12 w	⑧⑨
Hui Zhang,2018; China	Random number table method	30/30	EG:I 3 II 14 III 9 IV 4 CG:I 1 II 15 III 11 IV 3	N	Not reported	Erjialonggu(EJLG) Decoction+Endocrine therapy	Endocrine therapy+Oryzanol	2 w	①③④⑤⑥⑦⑧⑨
Jingru Song, 2019;China	Random number table method	27/28	EG:I 4 II 10 III 6 IV 7 CG:I 5 II 12 III 5 IV 6	Post-menopausal	EG: 51.44 ± 10.970 CG: 50.75 ± 12.477	Chaihujialonggumuli(CHJLGML) Decoction+AI	AI	12 w	①⑦
Juan Zhou,2020; China	Random number table method	36/36	N	N	EG: 53.00 ± 6.51 CG: 52.00 ± 5.44	Xiao yao an kun(XYAK) Decoction+Endocrine therapy	Endocrine therapy+placebo	8 w	②③④⑤⑥
Judith S. Jacobson et al.,2001; American	Random number table method	29/30	N	N	Not reported	Black Cohosh	placebo	8 w	⑨⑩⑪
Junwen Pei et al., 2019;China	Random number table method	30/30	EG:I 4 II 12 III 8 IV 6 CG: I 8 II10 III 7 IV 5	N	EG: 52.76 ± 8.0 CG: 55.66 ± 7.59	Danzhixiaoyao(DZXY) powder+Erxian(EX) Decoction+Endocrine therapy	Endocrine therapy	4 w	①⑦⑧⑨⑩
Junyuan Cai et al., 2021;China	Random number table method	30/30	N	Post-menopausal	EG: 61.2 ± 8.5 CG:61.9 ± 9.2	Fuzhengxiaoliu(FZXL) Decoction	Oryzanol+Vitamin B6	8 w	①⑧⑨⑩
Kaili Xu, 2019;China	Not reported	50/53	EG:I 20 II 30 CG:I 33 II 30	N	Not reported	Dangguiliuhuang(DGLH) Decoction+AI	AI	Not reported	①
Lan Luo et al., 2019;China	simple randomization	30/30	N	Premenopausal	EG: 44.07 ± 3.23 CG: 44.37 ± 3.08	JiaTCMibushen(JPBS) formula+Tamoxifen	Tamoxifen	4 w	①④⑤⑥⑪
Limin Zhu,2020; China	Random number table method	61/60	EG:II47 III 14 CG:II47 III13	Post-menopausal	EG: 59.43 ± 6.40 CG: 59.7 ± 6.61	Ruyanning(RYN) formula+AI	AI	8 w	①
Lina Sheng, 2014;China	Not reported	23/20	N	N	Not reported	Yangshenshugan(YSSG) Decoction+Endocrine therapy+Oryzanol+Vitamin B1	Endocrine therapy+Oryzanol+Vitamin B1	3 w	①⑦⑧⑨
Liujing Ou, 2018;China	Not reported	50/50	N	Post-menopausal	EG: 61.73 ± 5.9 CG: 61.66 ± 5.87	Bushenquyu (BSQY) formula+AI	AI	24 w	①⑦⑧⑪
Ming Feng et al.,2021	Interactive response technology	45/45	EG:I 15 II 30 CG:I 18 II 27	N	EG: 53.8 ± 7.04 CG:51.85 ± 7.84	Sanhuang(SH) Decoction	None	24w	①
Rongfei Jiang, 2021; China	Not reported	29/29	N	N	EG: 51.89 ± 9.97 CG: 55.89 ± 10.58	Chaihujialonggumuli(CHJLGML) Decoction+Endocrine therapy	Endocrine therapy	8 w	①
Rui Qiang, 2020;China	Random number table method	36/36	N	Post-menopausal	EG: 64.3 ± 5.5 CG: 63.7 ± 5.9	Banxiaxiexin(BXXX) Decoction+Endocrine therapy	Endocrine therapy	2 w	⑧⑨⑩
Ruiqing Sui, 2019;China	Not reported	29/30	N	Post-menopausal	Not reported	Zishuihanmu(ZSHM) formula+Endocrine therapy	Endocrine therapy	8 w	①②③⑥
Suzhen Lin et al., 2016;China	Not reported	34/34	N	Premenopausal	Not reported	Yiguan(YG) Decoction+Tamoxifen	Tamoxifen	12 w	②⑤
Ting Li et al., 2020;China	Not reported	36/36	N	N	EG: 51.88 ± 10.10 CG: 47.69 ± 10.73	Xiaoyaoankun(XYAK) Decoction+Endocrine therapy	Endocrine therapy+Placebo	8 w	②③④⑤⑥⑧⑨
Weikang Zhu,2020; China	Not reported	40/40	N	Post-menopausal	EG: 64.25 ± 5.67 CG: 60.28 ± 5.88	Bushenshugan(BSSG) formula+Calcium tablet	Calcium tablet	24 w	①⑦⑧⑨⑩⑪
Xiaoling Wang, 2018;China	Not reported	30/30	N	Premenopausal	EG: 40.66 ± 6.87 CG: 40.73 ± 6.9	TCM + Oryzanol	Oryzanol	12 w	①⑦⑧⑨⑩⑪
Xiaomei Wu et al., 2021; China	Random number table method	39/39	EG:I 5 II 14 III 13 IV 7 CG:I 6 II 14 III 13 IV 5	N	EG: 53.44 ± 5.49 CG: 53.38 ± 5.74	TCM + Oryzanol+Tamoxifen+Vitamin B1	Oryzanol+Tamoxifen+Vitamin B1	12w	⑧⑨⑩
Xiaozhen Liang, 2011; China	Not reported	40/40	N	Premenopausal	EG: 47.5 CG: 47	Liuweidihuang(LWDH) Decoction+Oryzanol	Oryzanol	8 w	⑦
Yang Fu, 2016; China	Random number table method	50/50	EG:I ~ II 26 III ~ IV 24 CG:I ~ II24 III ~ IV 26	N	EG: 45.1 ± 12.2 CG: 42.9 ± 13.8	Heixiaoyao(HXY) Powder+Shensiwei(SSW) Decoction+Tamoxifen	Tamoxifen+Oryzanol+Vitamin B1 + Vitamin B6	16 w	①⑧⑨
Yemei Li et al., 2022; China	Not reported	25/25	EG:I 7 II11 III 7 CG:I 8 II10 III 7	N	EG: 53.76 ± 8.86 CG: 54.96 ± 8.55	Jiaweifangjidihuang(JWFJDH) Decoction	Alprazolam	2 w	①
Yining Song et al., 2014;China	Not reported	60/60	N	N	Not reported	Black Cohosh	placebo	12 w	①
Zhihui Tao et al., 2020;China	Random number table method	30/30	EG:I ~ II 24 III ~ IV 6 CG:I ~ II 24 III ~ IV 26	N	EG: 50.263 ± 5.230 CG:51.566 ± 7.0353	Yishenkangai(YSKA) formula+Endocrine therapy	Endocrine therapy+Oryzanol	8 w	①⑦
Zhipei Han et al., 2021; China	Not reported	40/40	EG:I 1 II 20 III 15 IV 4 CG:I 2 II 14 III 18 IV 6	Post-menopausal	EG: 58.93 ± 7.15 CG: 58.80 ± 7.13	Ruyanning(RYN) formula+AI	AI	8 w	①⑧⑨⑩
Ziyi Yan, 2021;China	Random number table method	28/28	EG:I 4 II 19 III 5 CG:I 8 II 16 III 4	N	Not reported	Qingxinzishen(QXZS) Decoction+Endocrine therapy	Endocrine therapy	12 w	①②③④⑥⑧⑨

①: Total of KMI; ②: Hot flashes and night sweats in KMI; ③: Paraesthesia in KMI; ④: Osteoarthralgia in KMI; ⑤: Anxiety in KMI; ⑥: Insomnia in KMI; ⑦: objective response rate (ORR); ⑧: E2; ⑨: FSH; ⑩: LH; ⑪: Adverse Events (AEs). *CG* control group, *EG* experimental group, *N* not reported

### Quality evaluation of the literature

**Fig. 2:**
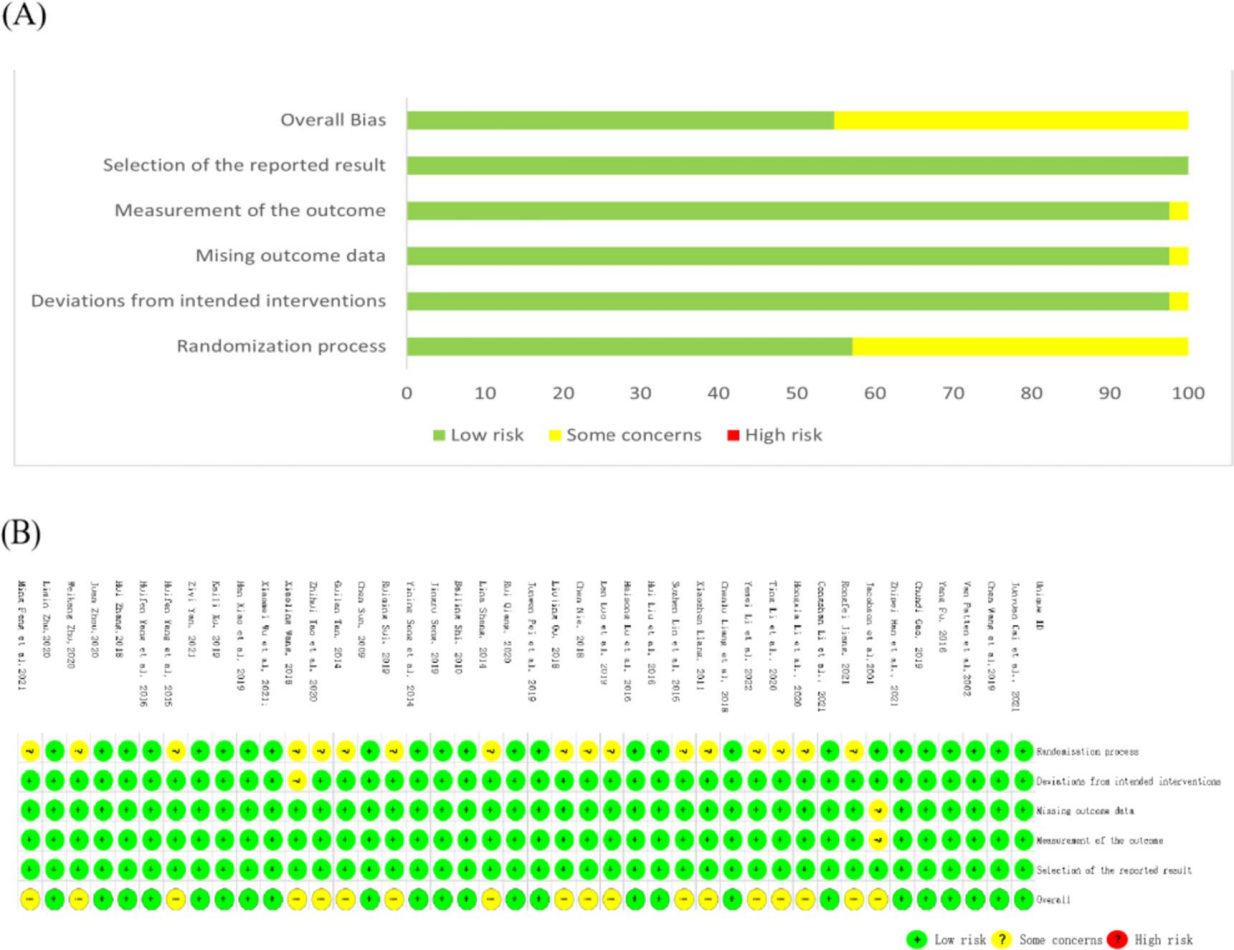
Risk of bias of included studies. (A) Risk of bias graph; (B) Risk of bias summary

The quality of the included studies was assessed using the Cochrane Collaboration RoB 2.0 tool (Fig. 2). Regarding random sequence generation, 25 RCTs were evaluated as having “some concerns” because these studies only mentioned the randomized grouping but did not mention the specific allocation methods used. Four RCTs used other randomized methods, which could lead to random bias. One RCT may have had a bias of ‘Deviations from intended interventions’. One RCT may have had a bias of ‘Measurement of the outcome’. The risk of selection and reporting bias were low in all the studies.

### Outcomes

#### KMI

**Fig. 3:**
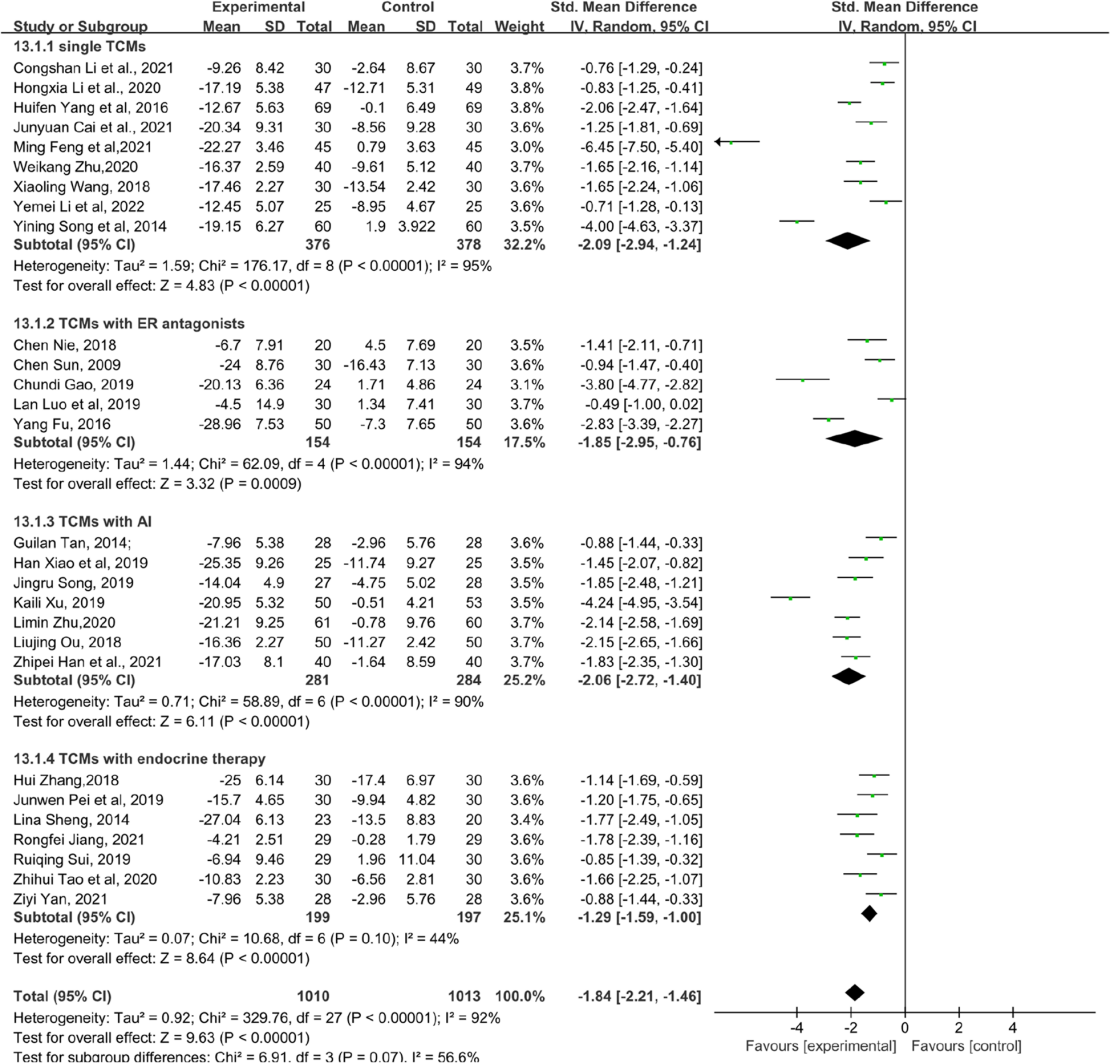
Forest plot showing the total KMI score (CI = confidence interval; SMD = standardized MD)

Twenty-eight RCTs used Kupperman (KMI) to evaluate MLS. The total KMI score in the experimental groups that received TCM was significantly lower than that in the control groups (standardized MD, SMD = − 1.84, 95% confidence interval, CI [− 2.21–-1.46], Z = 9.63, *P* < 0.00001). Owing to the high clinical heterogeneity and low quality of the included literature, some heterogeneity occurred. (*I*^2^ = 92%) (Fig. 3). Reviewing the included article data, it was found that 5 RCTs used KMI tables with different versions, which had an impact on the heterogeneity. After conducting sensitivity analysis, heterogeneity decreased to 74% (standardized MD, SMD = − 1.38, 95% confidence interval, CI [− 1.61–-1.16], Z = 11.96, *P* < 0.00001). The sensitivity analysis image can be found in Supplementary file 5.

**Fig. 4:**
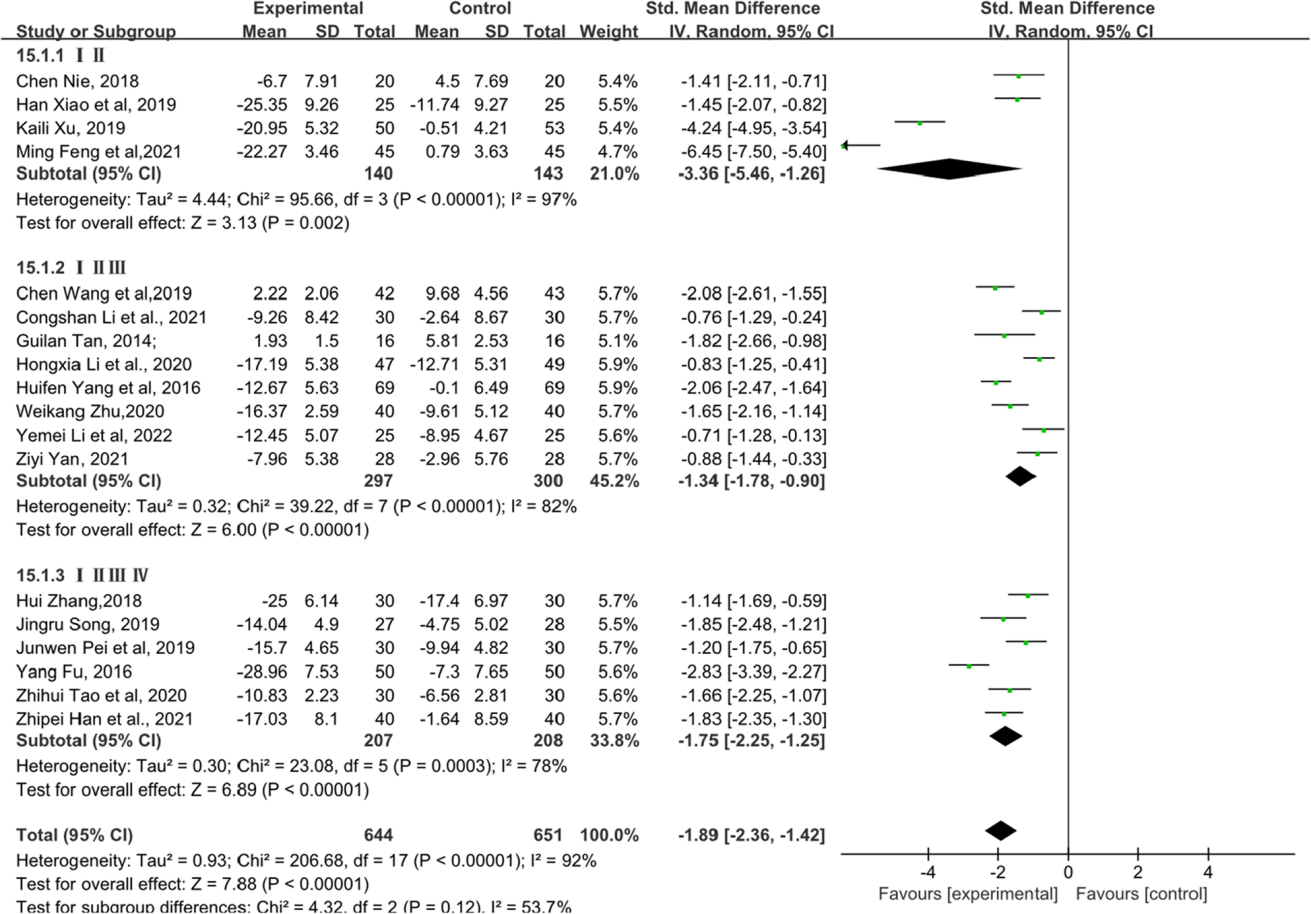
Forest plot showing the total KMI score based on tumour stage

In addition, it was interesting to compare the efficacy and safety of TCM for the treatment of MLS in different stages of BC. Because the inclusion of RCTs did not provide a hierarchical design for different tumour stages, this article can only display the forest plot showing the total KMI score based on stages of BC, as shown in Fig. 4. The results showed that the TCM group was better at decreasing the KMI scores (standardized MD, SMD = − 1.85, 95% confidence interval, CI [− 2.29 – -1.4], Z = 8.13, *P* < 0.00001). In addition, 2 RCTs used KMI tables with different versions, which had an impact on heterogeneity (standardized MD, SMD = − 1.5, 95% confidence interval, CI [− 1.8 – -1.21], Z = 9.92, *P* < 0.00001). The sensitivity analysis image can be found in Supplementary file 4.

**Fig. 5:**
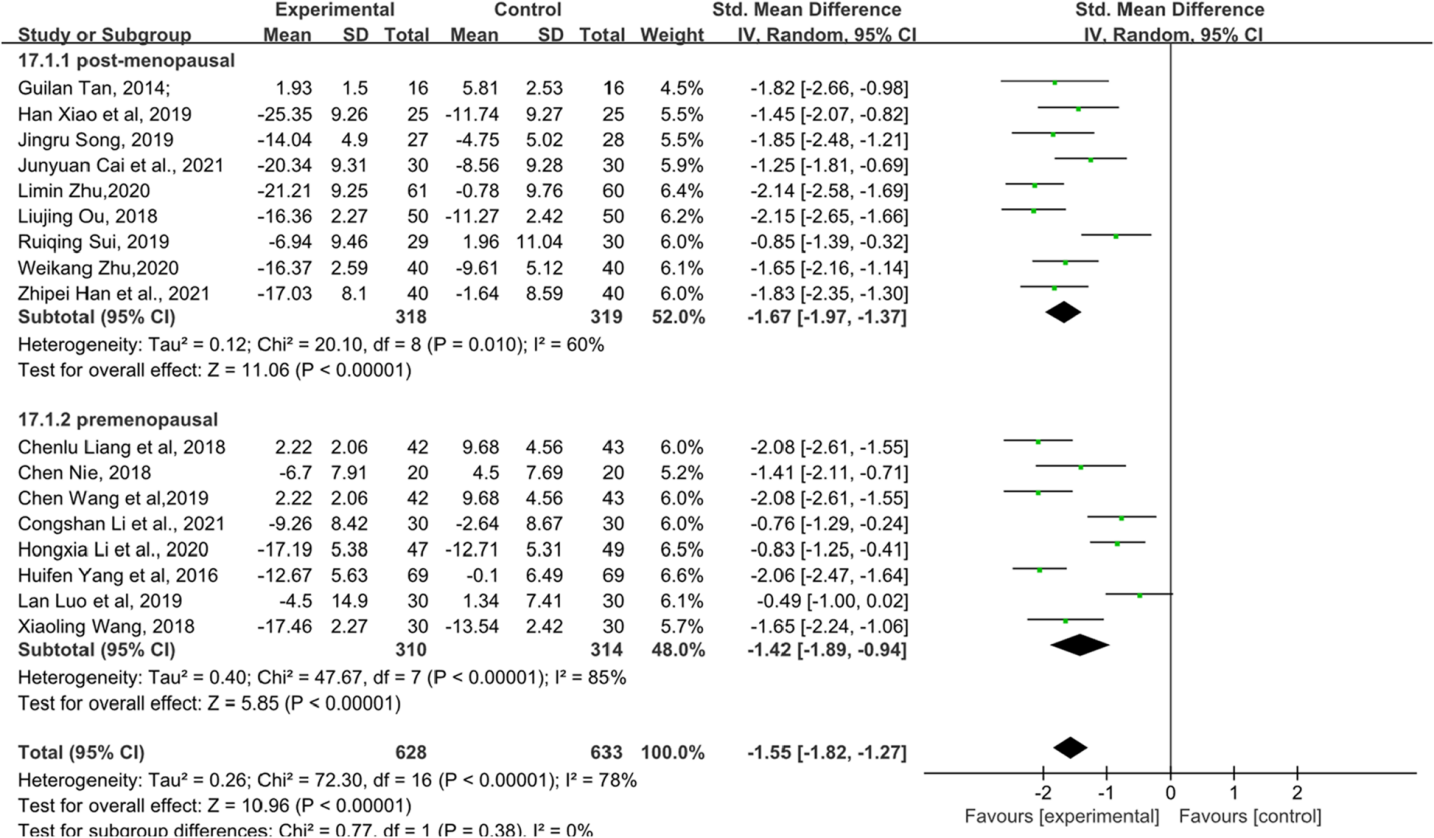
Forest plot showing the total KMI score based on the menstrual cycle

Due to the particularity of endocrine therapy for breast cancer, this article also analysed the KMI with the menstrual cycle as the subgroup analysis standard. As shown in Fig. 5, according to the grouping of menstrual cycles, each subgroup and overall data of the observation group showed better efficacy in treating MLS (standardized MD, SMD = − 1.55, 95% confidence interval, CI [− 1.82 – -1.27], Z = 10.96, *P* < 0.00001). The sensitivity analysis image can be found in Supplementary file 5.

**Fig. 6:**
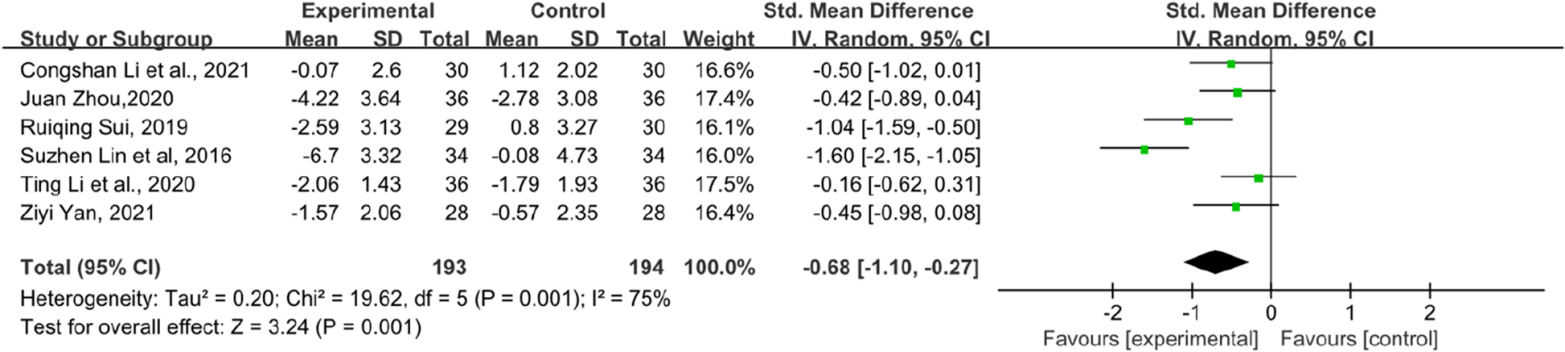
Forest plot showing hot flashes and night sweats in the KMI. (SMD = standardized MD)

**Fig. 7:**
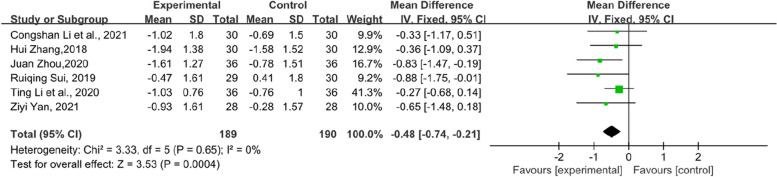
Forest plot of paraesthesia in KMI

**Fig. 8:**
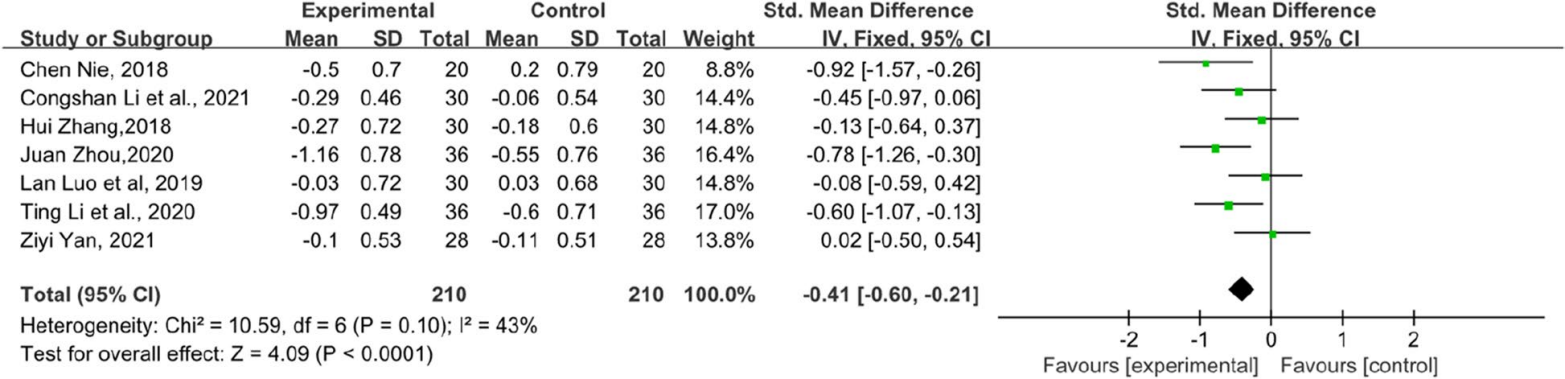
Forest plot of osteoarthralgia in KMI

**Fig. 9:**
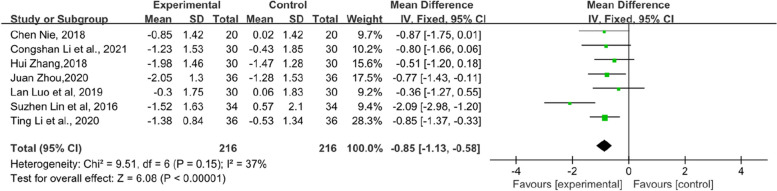
Forest plot of anxiety in KMI

**Fig. 10:**
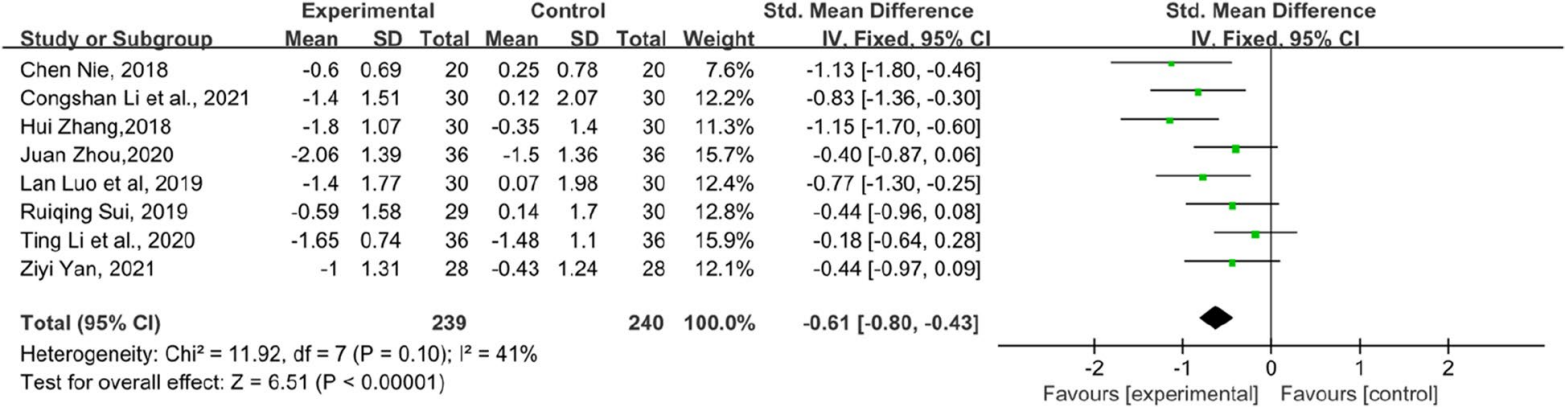
Forest plot of insomnia in KMI

In addition, the symptom scores of each item in the KMI score table also have statistical significance. Flashes of hot flashes and night sweats, which were some of the most significant symptoms of menopause, were analysed in 6 studies. Compared with the control group, the experimental group showed a significant relief in the outcome of hot flashes and night sweats in KMI (SMD = − 0.68, 95% CI [− 1.1–-0.27], Z = 3.24, *P* = 0.001). However, the heterogeneity was slightly higher (*I*^*2*^ = 75%). The results are shown in Fig. 6. Paraesthesia in KMI was reported by 6 RCTs (Fig. 7). The result suggests that the experimental group had an obvious effect on paraesthesia in KMI compared with the control group (SMD = − 0.48, 95% CI [− 0.74–-0.21], Z = 3.53, *P* = 0.0004). In addition, 7 RCTs reported osteoarthralgia among the KMI score table and showed that patients who received TCM tended to have better improvement in osteoarthralgia than did those who received endocrine therapy or placebo alone. (SMD = − 0.41, 95% CI [− 0.6–0.21], Z = 4.09, *P* < 0.0001). The results of the forest plot of osteoarthralgia in Kupperman are shown in Fig. 8. Moreover, 7 RCTs recorded anxiety in KMI. Compared with the control group, the experimental group had lower anxiety (MD = − 0.85, 95% CI [− 1.13, − 0.58], Z = 6.08, *P* < 0.00001) (Fig. 9). Eight RCTs showed insomnia in KMI. The findings indicated that the experimental group had a better effect than the control group (MD = − 0.61, 95% CI [− 0.8, − 0.43], Z = 6.51, *P* < 0.00001) (Fig. 10). From the data analysis, the TCM group plays an important role in alleviating the MLS of patients with BC after endocrine therapy.

#### Orr

**Fig. 11:**
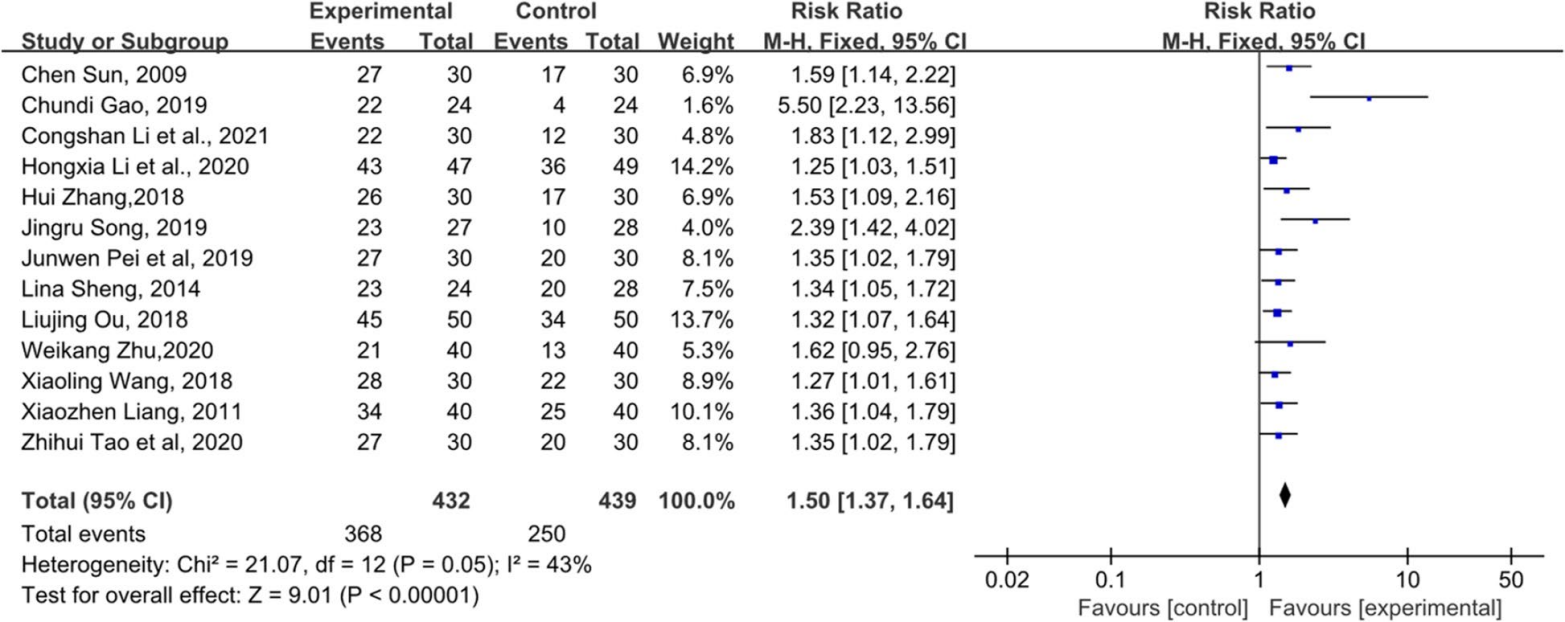
Forest plot of ORR

Thirteen studies mentioned ORR, and the meta-analysis indicated that the experimental group had a significant improvement in ORR compared with the control group (RR = 1.5, 95% CI [1.37–1.64], Z = 9.01, *P* < 0.00001). (Fig. 11).

### Female hormone

**Fig. 12:**
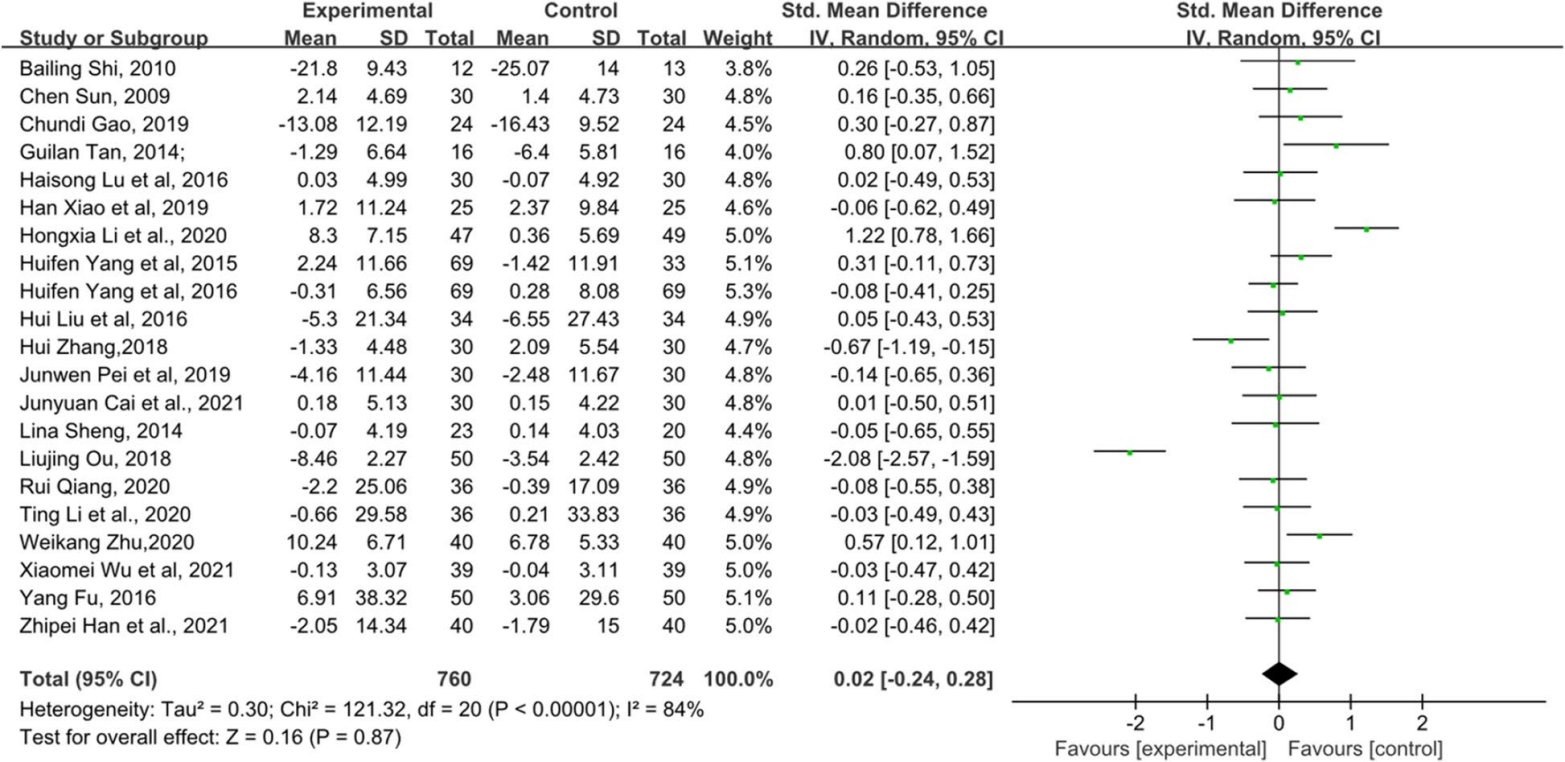
Forest plot of E_2_

**Fig. 13:**
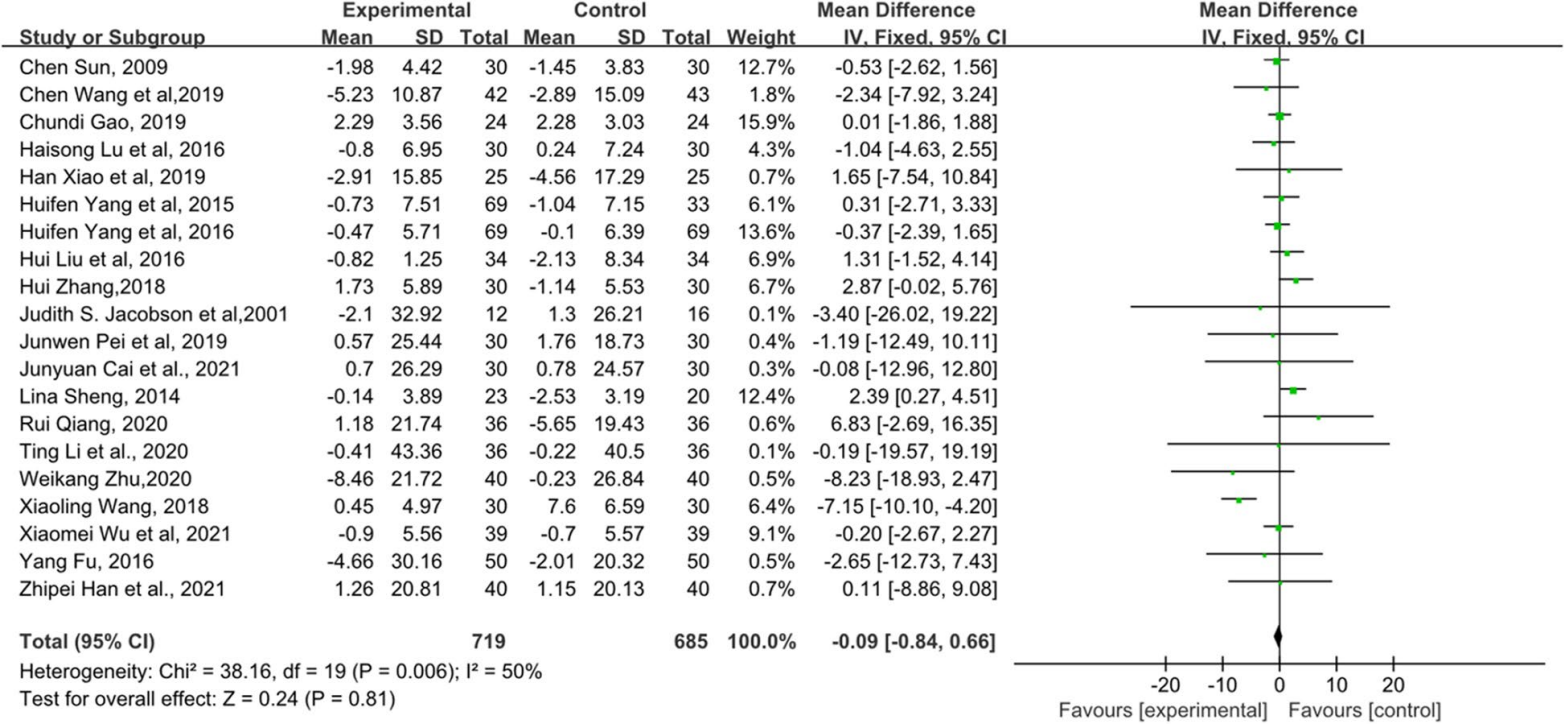
Forest plot of FSH

**Fig. 14:**
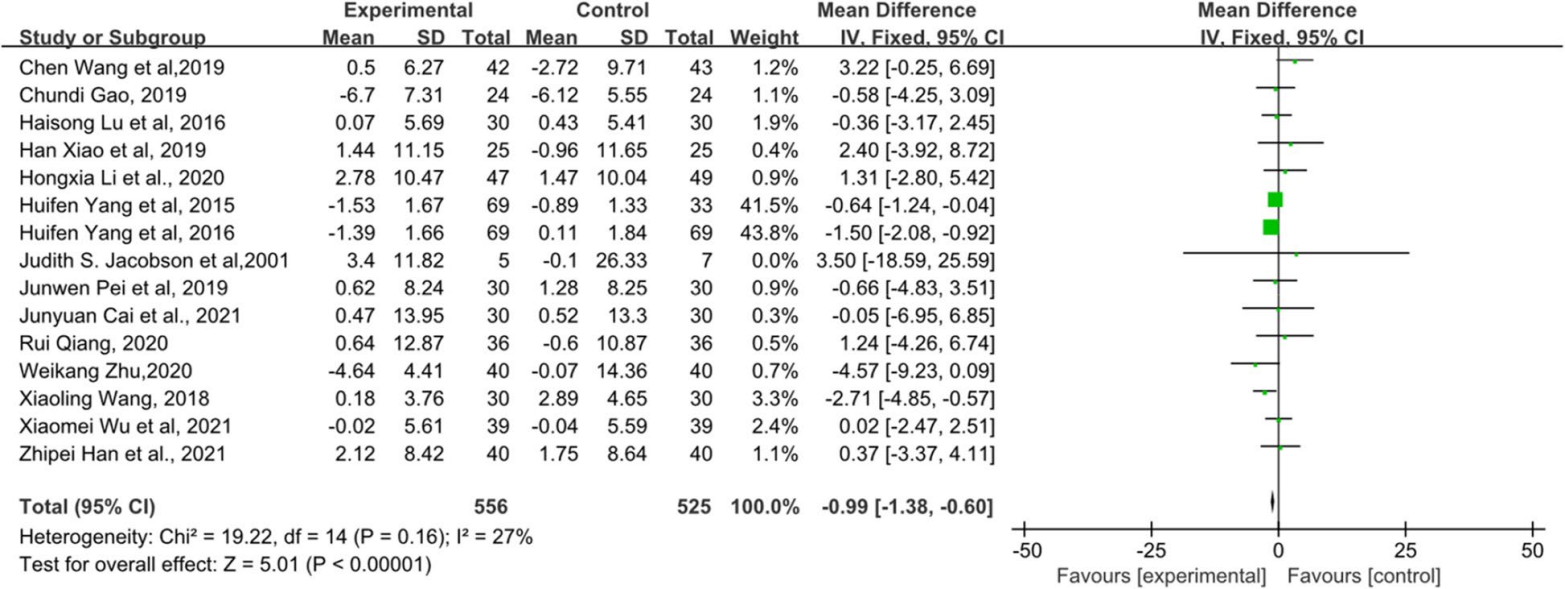
Forest plot of LH

Female hormones mainly include E_2_, FSH and LH. First, 21 RCTs, which included 1484 patients, mentioned E_2_ (Fig. 12). The results indicated that there was no statistical significance between the TCM groups and the control groups (*p* = 0.87). In addition, there were a total of 20 RCTs including 1404 patients related to FSH, and the forest plot of FSH also did not show a significant improvement between the two groups (*p* = 0.81) (Fig. 13). Fifteen RCTs comprising 1081 patients showed that those who received TCM tended to have a greater reduction in LH than those who received endocrine therapy or placebo in Fig. 14 (MD = − 0.99, 95% CI [− 1.38, − 0.5], Z = 5.01, *P* < 0.00001). The changes in female hormones suggest that TCM intervention will not affect the treatment of BC with endocrine drugs.

#### AEs

Six RCTs mentioned AEs, including menstrual disorder, arthralgia, hot flashes, elevated blood pressure, oedema and gastrointestinal discomfort. The RCTs showed that the incidence of AEs such as arthralgia and oedema in the intervention group was lower than that in the control group. No statistically significant differences were found in the occurrence rates of menstrual disorders, hot flashes, elevated blood pressure, or gastrointestinal discomfort. This indicates that TCM was safe in the treatment of Menopause-like Syndrome of BC.

### Analysis of publication Bias

**Fig. 15:**
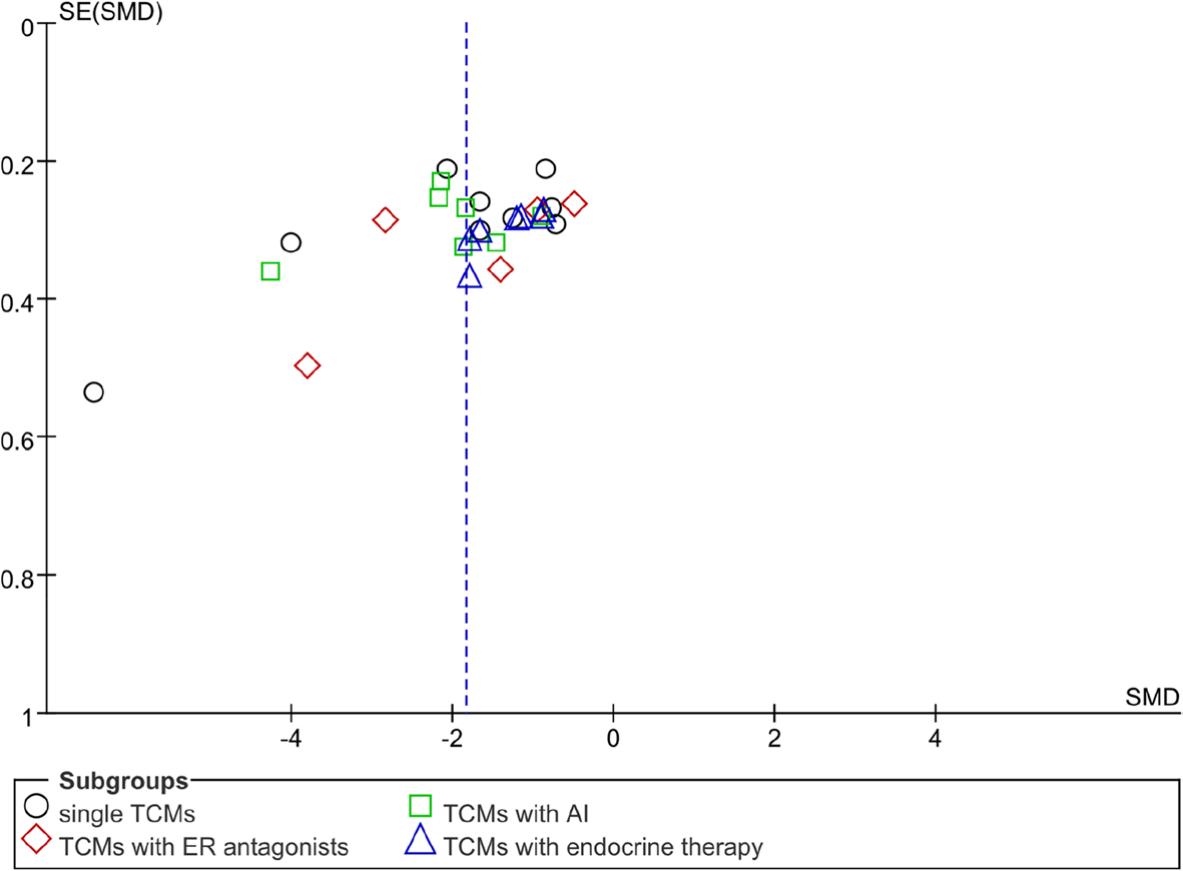
Funnel plot of KMI

**Fig. 16:**
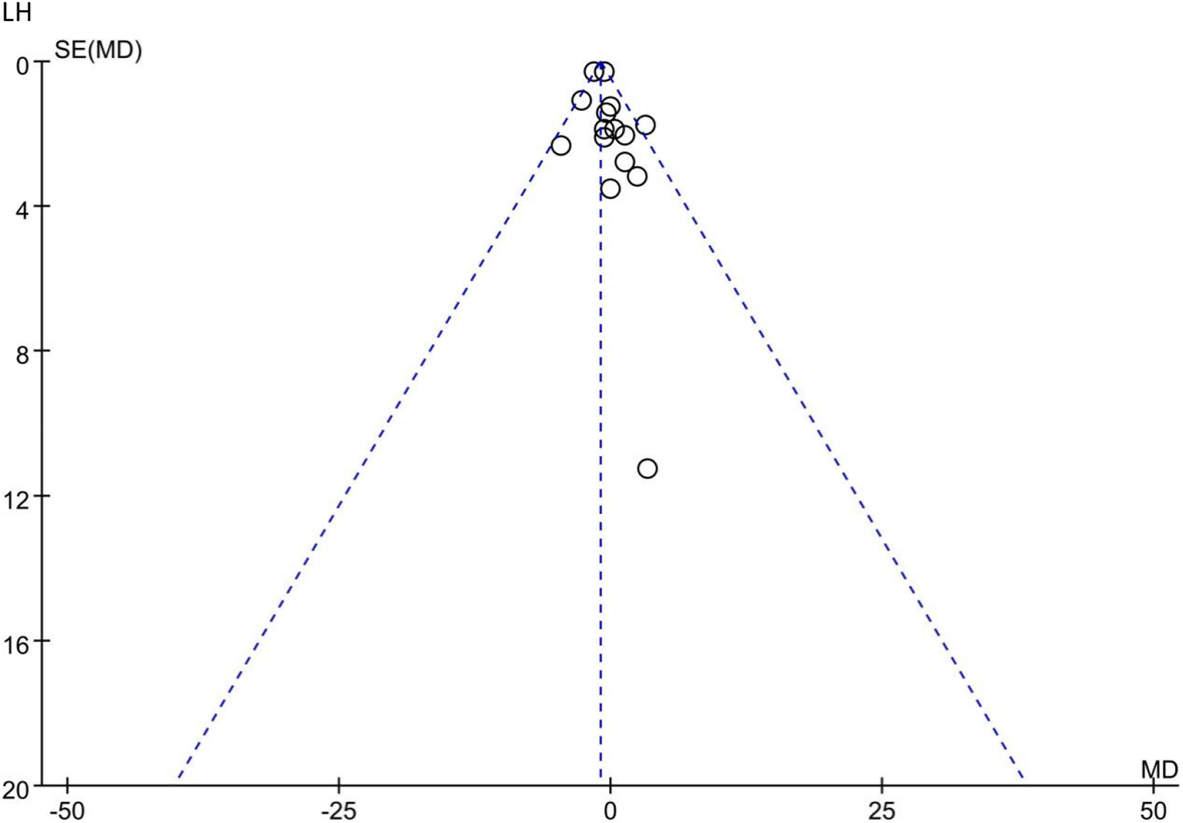
Funnel plot of FSH

**Fig. 17:**
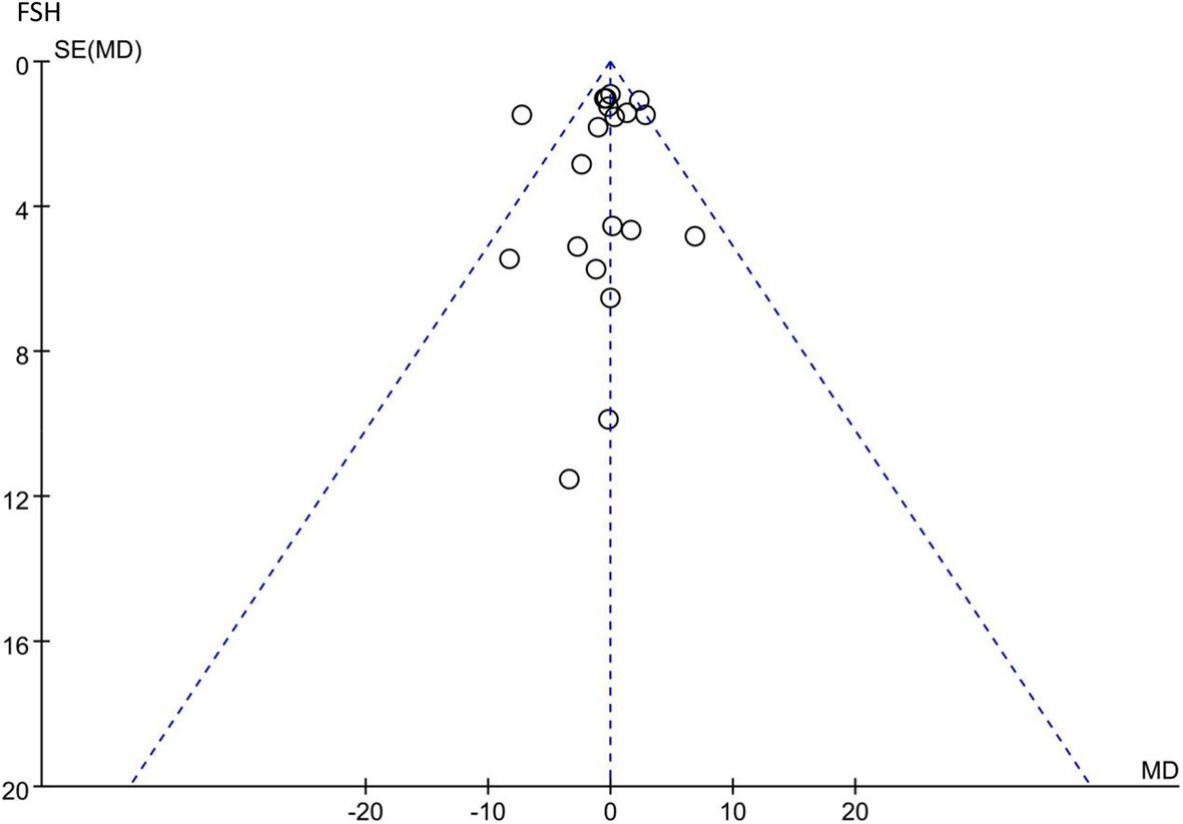
Funnel plot of LH

According to the funnel plot, KMI, FSH and LH were analysed. The funnel plot of the primary outcome (KMI) showed no complete symmetry and indicated the existence of publication bias. Publication bias may be associated with negative results not being published. The data distribution of FSH and LH was uneven, which indicated some publication bias. The results of the analysis of publication bias are shown in Figs. 15, 16, and 17.

### Assessment of the quality of evidence

Based on the GRADE criteria, the quality of evidence assessment was performed (Table 2). Most of the total KMI, MLS symptoms of each item in KMI, ORR, female hormones and AEs were assessed as low-quality evidence owing to clinical heterogeneity and low participant numbers in most studies. The quality of evidence assessment is available in Supplementary file 5.

**Table 2:** Quality of evidence assessment

Participants (studies) Follow-up	Risk of bias	Inconsistency	Indirectness	Imprecision	Publication bias	Overall certainty of evidence	Study event rates (%)		Relative effect (95% CI)	Anticipated absolute effects	
							With Treatment of BC	With TCM		Risk with Treatment of BC	Risk difference with TCM
**Total score of KMI**											
2023 (28 RCTs)	serious ^a^	not serious	not serious	not serious	none	⨁⨁⨁◯ Moderate	1010	1013	–	–	S MD **1.84 SD lower** (2.21 lower to 1.46 lower)
**Hot flashes and night sweats in KMI**											
387 (6 RCTs)	serious ^a^	serious ^b^	not serious	serious ^c^	none	⨁◯◯◯ Very low	194	193	–	–	S MD **0.68 SD lower** (1.1 lower to 0.27 lower)
**Paresthesia in KMI**											
379 (6 RCTs)	serious ^a^	not serious	not serious	serious ^c^	none	⨁⨁◯◯ Low	190	189	–		MD **0.48 lower** (0.74 lower to 0.21 lower)
**Osteoarthralgia in KMI**											
420 (7 RCTs)	serious ^a,d^	serious ^b^	not serious	not serious	none	⨁⨁◯◯ Low	210	210	–	–	S MD **0.41 SD lower** (0.6 lower to 0.21 lower)
**Anxiety in KMI**											
432 (7 RCTs)	serious ^a^	not serious	not serious	not serious	none	⨁⨁⨁◯ Moderate	216	216	–		MD **0.85 lower** (1.13 lower to 0.58 lower)
**Insomnia in KMI**											
479 (8 RCTs)	serious ^a,d^	serious ^b^	not serious	not serious	none	⨁⨁◯◯ Low	240	239	–	–	S MD **0.61 SD lower** (0.8 lower to 0.43 lower)
**ORR**											
871 (13 RCTs)	not serious	serious ^b^	not serious	not serious	none	⨁⨁⨁◯ Moderate	250/439 (56.9%)	368/432 (85.2%)	not es timable	569 per 1000	
**E**_**2**_											
1484 (21 RCTs)	serious ^a,d^	very serious ^b^	not serious	not serious	none	⨁◯◯◯ Very low	724	760	–	–	S MD **0.02 SD higher** (0.24 lower to 0.28 higher)
**FSH**											
1404 (20 RCTs)	serious ^d^	serious ^b^	not serious	not serious	none	⨁⨁◯◯ Low	685	719	–		MD **0.09 SD lower** (0.84 lower to 0.66 higher)
**LH**											
1081 (15 RCTs)	serious ^d^	serious ^b^	not serious	not serious	none	⨁⨁◯◯ Low	525	556	–		**MD 0.99 lower** (1.38 lower to 0.6 lower)
**AEs**											
1186 (6 RCTs)	not serious	serious ^b^	not serious	not serious	none	⨁⨁⨁◯ Moderate	67/598 (11.2%)	45/588 (7.7%)	not es timable	112 per 1000	

*CI* confidence interval, *MD* mean difference, *RR* risk ratio, *SMD* standardised mean difference

Explanations

^a^Most information is from studies at unclear risk of bias

^b^Clinical heterogeneity exists owing to the different treatment plan

^c^Small sample size

^d^Potential limitations are likely to lower confidence in the estimate of effect

## Discussion

In this study, an extensive search was performed to evaluate the efficacy and safety of TCM in the treatment of MLS for BC. On the one hand, this article mainly explains that TCM can improve patients’ quality of life and prolong survival time. On the other hand, the article also demonstrates that TCM does not change patients’ hormone levels, which cannot cause recurrence and metastasis. The specific performance is the relief of hot flashes and night sweats, paraesthesia, osteoarthralgia, anxiety and insomnia. In addition, common AEs, such as arthralgia and oedema, were significantly decreased in the TCM groups compared with the control groups. No statistically significant differences were found in the occurrence rates of menstrual disorders, hot flashes, elevated blood pressure, or gastrointestinal discomfort. This means that there were no differential AEs in the MLS with TCM treatment group compared to the control group.

BC has become a major health problem for women over the past two decades owing to its high incidence and mortality rates [22]. The epidemiology of BC in women shows that high levels of sex hormones lead to early menarche and late menopause in women, which has been proven to be related to the increased risk of BC [23]. A two-sample Mendelian randomization study found that increasing levels of bioavailable testosterone, dehydroepiandrosterone sulphate, testosterone, and oestradiol may increase the risk of ER + BC, consistent with the results of observational studies [24]. The use of aromatase inhibitors (AIs), LHRH-α, oestrogen receptor antagonists, and other adjuvant endocrine therapies in clinical practice is the standard treatment method for ER+ BC patients. Although endocrine therapy is available to BC patients, it has many adverse effects, such as causing MLS, which worsens the quality of life of patients and prognosis [25]. In addition, long-term use of endocrine therapy may lead to drug resistance. A study found that approximately 30% of BC patients will develop endocrine resistance after receiving tamoxifen treatment [26]. The latest research has found that when patients develop resistance to endocrine therapy, their sex hormone levels begin to fluctuate and gradually increase, leading to tumour metastasis or recurrence [27]. The acquired drug resistance of ER^+^ BC is a complex and dynamic biological process. Thus, it is critical to assess and treat MLS in BC survivors.

In recent years, TCM and their synthetic derivatives have made significant contributions to drug therapy, especially for cancers [6, 28]. One previous study summarized and discussed the effects of TCM in tumour treatment [29]. For instance, TCM can exert antitumour effects by inhibiting myeloid-derived suppressor cells, enhancing natural killer and cytolytic T cells [30], alleviating resistance to multiple chemotherapeutic drugs through their multitargeted therapeutic effects [31, 32], sensitizing tumour cells to chemotherapy drug s[33] and lessening the side effects of the rest of the treatmen t[34]. A clinical study found that TCM can safely and effectively improve MLS in BC patients. With the development of tumour treatment, TCM, which have been considered new potential modulators, have shown broad research prospects [11, 35, 36]. An increasing number of RCTs are using TCM to treat MLS in BC survivors.

Although the meta-analysis strictly followed the review procedures published, there are still some limitations that deserve to be explored for future improvement. First, although the RCTs included in this paper are sufficient, most of them are small sample tests, and the standards between the tests are not uniform, which may lead to high heterogeneity. Second, the heterogeneity of the data analysed by tumour staging and menstrual cycle subgroups in the article was relatively low, while the heterogeneity was higher when endocrine therapy drugs were used as the grouping standard. This may be due to the variety of endocrine therapy drugs and differences in drug use among manufacturers or individuals, resulting in inconsistent standards and high heterogeneity. Third, none of the trials showed allocation concealment or blinding procedures, which also reflects the problem of low quality of the article. It will be even better to collect high-quality evidence from papers. However, for the above three reasons, there is a lack of high-quality original research in this systematic review and meta-analysis. More RCTs with large-scale, multicentre, and uniform criteria are needed. However, compared with the previous literature, this review thoroughly summarizes the clinical findings of past RCTs related to TCM and systematically discusses the main beneficial effects of TCM in the treatment of MLS. These findings suggest that treatment with TCM may improve the QoL of patients with BC.

## Conclusion

This review indicates the efficacy and Safety of TCM Medicine in the Treatment of MLS for BC Survivors. More prospectively designed, large-size, and standard clinical trials are needed to confirm the present findings.

### Supplementary Information

**Additional file 1.** PRISMA checklist.

**Additional file 2.** Search steps.

**Additional file 3.** Summary of all the included trials.

**Additional file 4.** Sensitivity analysis images.

**Additional file 5.** Quality of evidence assessment.

## Data Availability

The original contributions presented in the study are included in the article/supplementary material, and further inquiries can be directed to the corresponding authors. Data openly available in a public repository. Please see the Data Availability section of the Author guidelines for more details.

## References

[1] World Health Organization Breast cancer. 2022. [https://www.who.int/news-room/fact-sheets/detail/breastcancer] Accessed on date 5 Sept. 2023.

[2] Cancer Surveillance Branch (CSU). (2020) International Agency for Research on Cancer. [https://gco.iarc.fr/] Accessed on date Sept. 6, 2023.

[3] National Institutes of Health. Breast cancer. 2022. [https://seer.cancer.gov/statfacts/html/breast.html] Accessed on date 6 December 2022

[4] China anti cancer association (CACA) (2021). Guidelines and Guidelines for Diagnosis and Treatment of Breast Cancer of the Chinese Anticancer Association. Chinese J Cancer..

[5] Harbeck N, Gnant M (2017). Breast cancer. Lancet..

[6] Rees M, Angioli R, Coleman RL, Glasspool R, Plotti F, Simoncini T (2020). European Menopause and Andropause Society (EMAS) and International Gynecologic Cancer Society (IGCS) position statement on managing the menopause after breast cancer: focus on menopausal symptoms and osteoporosis. Maturitas..

[7] Ramchand SK, Cheung YM, Yeo B, Grossmann M (2019). The effects of adjuvant endocrine therapy on bone health in women with breast cancer. J Endocrinol..

[8] Yu Y, Yin W, Yu ZH, Zhou YJ, Chi JR, Ge J, Cao XC (2019). miR-190 enhances endocrine therapy sensitivity by regulating SOX9 expression in breast cancer. J Exp Clin Cancer Res..

[9] Newman DJ, Cragg GM (2016). Natural products as sources of new drugs from 1981 to 2014. J Nat Prod..

[10] Afolabi LO, Bi J, Chen L, Wan X (2021). A natural product, piperlongumine (PL), increases tumor cells sensitivity to NK cell killing. Int Immunopharmacol..

[11] Deng LJ, Qi M, Li N, Lei YH, Zhang DM, Chen JX (2020). Natural products and their derivatives: promising modulators of tumor immunotherapy. J Leukoc Biol..

[12] Dias AS, Helguero L, Almeida CR, Duarte IF (2021). Natural compounds as metabolic modulators of the tumor microenvironment. Molecules..

[13] Focaccetti C, Izzi V, Benvenuto M, Fazi S, Ciuffa S, Giganti MG (2019). Polyphenols as immunomodulatory compounds in the tumor microenvironment: friends or foes?. Int J Mol Sci..

[14] Tripathy D, Im SA, Colleoni M (2018). Ribociclib plus endocrine therapy for premenopausal women with hormone-receptor-positive, advanced breast cancer (MONALEESA-7): a randomised phase 3 trial. Lancet Oncol..

[15] Toi M, Imoto S, Ishida T, Ito Y, Iwata H, Masuda N, Mukai H, Saji S, Shimizu A, Ikeda T, Haga H, Saeki T, Aogi K, Sugie T, Ueno T, Kinoshita T, Kai Y, Kitada M, Sato Y, Jimbo K, Sato N, Ishiguro H, Takada M, Ohashi Y, Ohno S (2021). Adjuvant S-1 plus endocrine therapy for oestrogen receptor-positive, HER2-negative, primary breast cancer: a multicentre, open-label, randomised, controlled, phase 3 trial. Lancet Oncol..

[16] Page MJ, Moher D, Bossuyt PM, Boutron I, Hoffmann TC, Mulrow CD (2021). PRISMA 2020 explanation and elaboration: updated guidance and exemplars for reporting systematic reviews. BMJ..

[17] Delaplaine RW, Bottomy JR, Blatt M (1952). Effective control of the surgical menopause by estradiol pellet implantation at the time of surgery. Surg Gynecol Obstet..

[18] Eisenhauer EA, Therasse P, Bogaerts J (2009). New response evaluation criteria in solid tumours: revised RECIST guideline (version 1.1). Eur J Cancer.

[19] Zhang L, Han K (2009). How to analyze change from baseline: Absolute or percentage change. D-level Essay in Statistics.

[20] Julian Higgins, James Thomas, et al. Cochrane Handbook for Systematic Reviews of Interventions. Version 6.3, 2022. Chapter 6: Choosing effect measures and computing estimates of effect | Cochrane Training. [https://training.cochrane.org/handbook/current/chapter-06-467.] Accessed on date Sept. 6, 2023.

[21] Higgins JP, Altman DG, Gøtzsche PC, Jüni P, Moher D, Oxman AD (2011). The Cochrane Collaboration’s tool for assessing risk of bias in randomised trials. BMJ..

[22] Loibl S, Poortmans P, Morrow M, Denkert C, Curigliano G (2021). Breast cancer. Lancet..

[23] Coughlin SS (2019). Epidemiology of Breast Cancer in Women. Adv Exp Med Biol..

[24] Nounu A, Kar SP, Relton CL (2022). Sex steroid hormones and risk of breast cancer: a two-sample Mendelian randomization study. Breast Cancer Res..

[25] Hernando C, Ortega-Morillo B, Tapia M (2021). Oral Selective Estrogen Receptor Degraders (SERDs) as a Novel Breast Cancer Therapy: Present and Future from a Clinical Perspective. Int J Mol Sci..

[26] Zeng Q, Lin X, Chen H (2022). Multi-time scale transcriptomic analysis on the dynamic process of tamoxifen resistance development in breast cancer cell lines. Breast Cancer..

[27] Cairns J, Ingle JN, Dudenkov TM (2020). Pharmacogenomics of aromatase inhibitors in postmenopausal breast cancer and additional mechanisms of anastrozole action. JCI Insight..

[28] Atanasov AG, Waltenberger B, Pferschy-Wenzig EM, et al. Discovery and resupply of pharmacologically active plant-derived natural products: a review. Biotechnol Adv. 33:1582–614. 10.1016/j.biotechadv.2015.08.001 PMC4748402 26281720

[29] Harvey AL, Edrada-Ebel R, Quinn RJ. The re-emergence of natural products for drug discovery in the genomics era. Nat Rev Drug Discov. 14:111–29. 10.1038/nrd4510 25614221

[30] Dutta S, Mahalanobish S, Saha S, et al. Natural products: an upcoming therapeutic approach to cancer. Food Chem Toxicol. 128:240–55. 10.1016/j.fct.2019.04.012 30991130

[31] Pan P, Huang YW, Oshima K, et al. The immunomodulatory potential of natural compounds in tumor-bearing mice and humans. Crit Rev Food Sci Nutr. 59:992–1007. 10.1080/10408398.2018.1537237 PMC6508979 30795687

[32] Guo Q, Cao H, Qi X, et al. Research progress in reversal of tumor multi-drug resistance via natural products. Anticancer Agents Med Chem. 17:1466–76. 10.2174/1871520617666171016105704 29034843

[33] Meerson A, Khatib S, Mahajna J. Natural products targeting cancer stem cells for augmenting cancer therapeutics. Int J Mol Sci. 22:13,044. 10.3390/ijms222313044 PMC8657727 34884848

[34] de Oliveira Júnior RG, Christiane Adrielly AF, da Silva Almeida JRG, et al. Sensitization of tumor cells to chemotherapy by natural products: A systematic review of preclinical data and molecular mechanisms. Fitoterapia. 129:383–400. 10.1016/j.fitote.2018.02.025 29476786

[35] Liu Y, Wang GC, Liu YJ, et al. Surgical concept and techniques of recurrent cervical cancer patients accompanied with high risk of intestinal obstruction after radical radiotherapy. Zhonghua Zhong Liu Za Zhi. 42:61–4. 10.3760/cma.j.issn.0253-3766.2020.01.009 32023771

[36] Fabiani R (2020). Antitumoral properties of natural products. Molecules..

