# Supplementary material for: Efficacy and safety of traditional Chinese medicine in the treatment of menopause-like syndrome for breast cancer survivors: a systematic review and meta-analysis
Source: BMC Cancer. 2024 Jan 8;24:42. doi: 10.1186/s12885-023-11789-z (PMC10773128; doi:10.1186/s12885-023-11789-z)
Supplement: Supplementary file 2 — Additional file 2. Search steps. [file 12885_2023_11789_MOESM2_ESM.pdf]

Table 1. Pubmed

Search steps	Search terms
#1	Breast Neoplasms[MeSH Major Topic]
#2	((((((((((((((((((((((((((((((((((((Breast Neoplasm[Title/Abstract]) OR (Neoplasm, Breast[Title/Abstract])) OR (Neoplasms, Breast[Title/Abstract])) OR (Breast Tumors[Title/Abstract])) OR (Breast Tumor[Title/Abstract])) OR (Tumor, Breast[Title/Abstract])) OR (Tumors, Breast[Title/Abstract])) OR (Breast Cancer[Title/Abstract])) OR (Cancer, Breast[Title/Abstract])) OR (Malignant Tumor of Breast[Title/Abstract])) OR (Breast Malignant Tumor[Title/Abstract])) OR (Breast Malignant Tumors[Title/Abstract])) OR (Cancer of the Breast[Title/Abstract])) OR (Cancer of Breast[Title/Abstract])) OR (Malignant Neoplasm of Breast[Title/Abstract])) OR (Breast Malignant Neoplasm[Title/Abstract])) OR (Breast Malignant Neoplasms[Title/Abstract])) OR (Mammary Cancer[Title/Abstract])) OR (Cancer, Mammary[Title/Abstract])) OR (Cancers, Mammary[Title/Abstract])) OR (Mammary Cancers[Title/Abstract])) OR (Mammary Carcinoma, Human[Title/Abstract])) OR (Carcinoma, Human Mammary[Title/Abstract])) OR (Carcinomas, Human Mammary[Title/Abstract])) OR (Human Mammary Carcinomas[Title/Abstract])) OR (Mammary Carcinomas, Human[Title/Abstract])) OR (Human Mammary Carcinoma[Title/Abstract])) OR (Mammary Neoplasms, Human[Title/Abstract])) OR (Human Mammary Neoplasm[Title/Abstract])) OR (Human Mammary Neoplasms[Title/Abstract])) OR (Neoplasm, Human Mammary[Title/Abstract])) OR (Neoplasms, Human Mammary[Title/Abstract])) OR (Mammary Neoplasm, Human[Title/Abstract])) OR (Breast Carcinoma[Title/Abstract])) OR (Breast Carcinomas[Title/Abstract])) OR (Carcinoma, Breast[Title/Abstract])) OR (Carcinomas, Breast[Title/Abstract])
#3	Biological Products[MeSH Major Topic] OR Medicine, Chinese Traditional[MeSH Major Topic] OR Drugs, Chinese Herbal[MeSH Major Topic] OR Herbal Medicine[MeSH Major Topic] OR Drugs, Chinese Herbal[MeSH Major Topic] OR Medicine, Chinese Traditional[MeSH Major Topic] OR Biological Products[MeSH Major Topic] OR Medicine, Chinese Traditional[MeSH Major Topic] OR Drugs, Chinese Herbal[MeSH Major Topic] OR Herbal Medicine[MeSH Major Topic]
#4	(Products, Biological[Title/Abstract])OR (Biological Product[Title/Abstract]) OR (Product, Biological[Title/Abstract]) OR (Biologic Product[Title/Abstract]) OR (Product, Biologic[Title/Abstract]) OR (Biologic Products[Title/Abstract]) OR (Biopharmaceuticals[Title/Abstract]) OR (Biopharmaceutical[Title/Abstract]) OR (Biological[Title/Abstract]) OR (Biologic[Title/Abstract]) OR (Biological Drug[Title/Abstract]) OR (Drug, Biological[Title/Abstract]) OR (Biologic Drugs[Title/Abstract]) OR (Drugs, Biologic[Title/Abstract]) OR (Biological Medicine[Title/Abstract]) OR (Medicine, Biological[Title/Abstract]) OR (Biological Medicines[Title/Abstract]) OR (Medicines, Biological[Title/Abstract]) OR (Biologicals[Title/Abstract]) OR (Biologic Medicines[Title/Abstract]) OR (Medicines,

	Biologic[Title/Abstract]) OR (Biologic Pharmaceuticals[Title/Abstract]) OR (Pharmaceuticals, Biologic[Title/Abstract]) OR (Biologics[Title/Abstract]) OR (Biologic Drug[Title/Abstract]) OR (Drug, Biologic[Title/Abstract]) OR (Biological Drugs[Title/Abstract]) OR (Drugs, Biological[Title/Abstract]) OR (Natural Products[Title/Abstract]) OR (Natural Product[Title/Abstract]) OR (Product, Natural[Title/Abstract]) OR (Medicine, Chinese Traditional[Title/Abstract]) OR (Traditional Chinese Medicine[Title/Abstract]) OR (Traditional Medicine, Chinese[Title/Abstract]) OR (Zhong Yi Xue[Title/Abstract]) OR (Chinese Traditional Medicine[Title/Abstract]) OR (Chinese Medicine, Traditional) OR((Drugs, Chinese Herbal[Title/Abstract]) OR (Chinese Drugs, Plant[Title/Abstract]) OR (Chinese Herbal Drugs[Title/Abstract]) OR (Herbal Drugs, Chinese[Title/Abstract]) OR (Plant Extracts, Chinese[Title/Abstract]) OR (Chinese Plant Extracts[Title/Abstract]) OR (Extracts, Chinese Plant[Title/Abstract]) OR (Herbal Medicine[Title/Abstract])
#5	Menopause[MeSH Major Topic] OR Perimenopause[MeSH Major Topic]
#6	((climacteric[Title/Abstract]) OR (menopause[Title/Abstract])) OR (kupperman[Title/Abstract])
#7	#1 OR #2
#8	#3 OR #4
#9	#5 OR #6
#10	#7 AND #8 AND #9
#11	#10 AND ("randomized controlled trial"[pt] OR "controlled clinical trial"[pt] OR randomized[tiab] OR placebo[tiab] OR "drug therapy"[sh] OR randomly[tiab] OR trial[tiab] OR groups[tiab])

Table 2. Web of science

Search steps	Search terms
#1	(((((((((((((((((((((((((((((((((((((TS=(Breast Neoplasms)) OR TS=(Breast Neoplasm)) OR TS=(Neoplasm, Breast)) OR TS=(Neoplasms, Breast)) OR TS=(Breast Tumors)) OR TS=(Breast Tumor)) OR TS=(Tumor, Breast)) OR TS=(Tumors, Breast)) OR TS=(Breast Cancer)) OR TS=(Cancer, Breast)) OR TS=(Malignant Tumor of Breast)) OR TS=(Breast Malignant Tumor)) OR TS=(Breast Malignant Tumors)) OR TS=(Cancer of the Breast)) OR TS=(Cancer of Breast)) OR TS=(Malignant Neoplasm of Breast)) OR TS=(Breast Malignant Neoplasm)) OR TS=(Breast Malignant Neoplasms)) OR TS=(Mammary Cancer)) OR TS=(Cancer, Mammary)) OR TS=(Cancers, Mammary)) OR TS=(Mammary Cancers)) OR TS=(Mammary Carcinoma, Human)) OR TS=(Carcinoma, Human Mammary)) OR TS=(Carcinomas, Human Mammary)) OR TS=(Human Mammary Carcinomas)) OR TS=(Mammary Carcinomas, Human)) OR TS=(Human Mammary Carcinoma)) OR TS=(Mammary Neoplasms, Human)) OR TS=(Human Mammary Neoplasm)) OR TS=(Human Mammary Neoplasms)) OR TS=(Neoplasm, Human Mammary)) OR TS=(Neoplasms, Human Mammary)) OR TS=(Mammary Neoplasm, Human)) OR TS=(Breast Carcinoma)) OR TS=(Breast Carcinomas)) OR TS=(Carcinoma, Breast)) OR TS=(Carcinomas, Breast)

#2	TS=(Biological Products) OR TS=(Medicine, Chinese Traditional) OR TS=(Drugs, Chinese Herbal) OR TS=(Herbal Medicine)
#3	((TS=Products, Biological)) OR TS=(Biological Product)) OR TS=(Product, Biological)) OR TS=(Biologic Product)) OR TS=(Product, Biologic)) OR TS=(Biologic Products)) OR TS=(Biopharmaceuticals)) OR TS=(Biopharmaceutical)) OR TS=(Biological)) OR TS=(Biologic)) OR TS=(Biological Drug)) OR TS=(Drug, Biological)) OR TS=(Biologic Drugs)) OR TS=(Drugs, Biologic)) OR TS=(Biological Medicine)) OR TS=(Medicine, Biological)) OR TS=(Biological Medicines)) OR TS=(Medicines, Biological)) OR TS=(Biologicals)) OR TS=(Biologic Medicines)) OR TS=(Medicines, Biologic)) OR TS=(Biologic Pharmaceuticals)) OR TS=(Pharmaceuticals, Biologic)) OR TS=(Biologics)) OR TS=(Biologic Drug)) OR TS=(Drug, Biologic)) OR TS=(Biological Drugs)) OR TS=(Drugs, Biological)) OR TS=(Natural Products)) OR TS=(Natural Product)) OR TS=(Product, Natural)) OR TS=(Medicine, Chinese Traditional)) OR TS=(Traditional Chinese Medicine)) OR TS=(Traditional Medicine, Chinese)) OR TS=(Zhong Yi Xue)) OR TS=(Chinese Traditional Medicine)) OR TS=(Chinese Medicine, Traditional) OR(TS=(Drugs, Chinese Herbal)) OR TS=(Chinese Drugs, Plant)) OR TS=(Chinese Herbal Drugs)) OR TS=(Herbal Drugs, Chinese)) OR TS=(Plant Extracts, Chinese)) OR TS=(Chinese Plant Extracts)) OR TS=(Extracts, Chinese Plant)) OR TS= (Herbal Medicine))
#4	TS=(Perimenopause) OR TS=(Menopause) OR TS=(climacteric) OR TS=(menopause) OR TS=(kupperman)
#5	#3 OR #2
#6	#5 AND #4 AND #1

Table 3. cochrane library

Search steps	Search terms
#1	MeSH descriptor: [Breast Neoplasms] explode all trees
#2	(Breast Neoplasm):ti,ab,kw OR (Neoplasm, Breast):ti,ab,kw OR (Neoplasms, Breast):ti,ab,kw OR (Breast Tumors):ti,ab,kw OR (Breast Tumor):ti,ab,kw OR (Tumor, Breast):ti,ab,kw OR (Tumors, Breast):ti,ab,kw OR (Breast Cancer):ti,ab,kw OR (Cancer, Breast):ti,ab,kw OR (Malignant Tumor of Breast):ti,ab,kw OR (Breast Malignant Tumor):ti,ab,kw OR (Breast Malignant Tumors):ti,ab,kw OR (Cancer of the Breast):ti,ab,kw OR (Cancer of Breast):ti,ab,kw OR (Malignant Neoplasm of Breast):ti,ab,kw OR (Breast Malignant Neoplasm):ti,ab,kw OR (Breast Malignant Neoplasms):ti,ab,kw OR (Mammary Cancer):ti,ab,kw OR (Cancer, Mammary):ti,ab,kw OR (Cancers, Mammary):ti,ab,kw OR (Mammary Cancers):ti,ab,kw OR (Mammary Carcinoma, Human):ti,ab,kw OR (Carcinoma, Human Mammary):ti,ab,kw OR (Carcinomas, Human Mammary):ti,ab,kw OR (Human Mammary Carcinomas):ti,ab,kw OR (Mammary Carcinomas, Human):ti,ab,kw OR (Human Mammary Carcinoma):ti,ab,kw OR (Mammary Neoplasms, Human):ti,ab,kw OR (Human Mammary Neoplasm):ti,ab,kw OR (Human Mammary Neoplasms):ti,ab,kw OR (Neoplasm, Human Mammary):ti,ab,kw OR (Neoplasms, Human Mammary):ti,ab,kw OR (Mammary Neoplasm, Human):ti,ab,kw OR (Breast Carcinoma):ti,ab,kw OR (Breast Carcinomas):ti,ab,kw OR (Carcinoma, Breast):ti,ab,kw OR (Carcinomas, Breast):ti,ab,kw
#3	MeSH descriptor: [Biological Products] explode all trees

#4	MeSH descriptor: [Medicine, Chinese Traditional] explode all trees
#5	MeSH descriptor: [Drugs, Chinese Herbal] explode all trees
#6	MeSH descriptor: [Herbal Medicine] explode all trees
#7	(Traditional Chinese Medicine):ti,ab,kw OR (Traditional Medicine, Chinese):ti,ab,kw OR (Zhong Yi Xue):ti,ab,kw OR (Chinese Traditional Medicine):ti,ab,kw OR (Chinese Medicine, Traditional):ti,ab,kw OR (Chinese Drugs, Plant):ti,ab,kw OR (Chinese Herbal Drugs):ti,ab,kw OR (Herbal Drugs, Chinese):ti,ab,kw OR (Plant Extracts, Chinese):ti,ab,kw OR (Chinese Plant Extracts):ti,ab,kw OR (Extracts, Chinese Plant):ti,ab,kw OR (Herbal Medicine):ti,ab,kw OR (Products, Biological):ti,ab,kw OR (Biological Product):ti,ab,kw OR (Product, Biological):ti,ab,kw OR (Biologic Product):ti,ab,kw OR (Product, Biologic):ti,ab,kw OR (Biologic Products):ti,ab,kw OR (Biological):ti,ab,kw OR (Biologic):ti,ab,kw OR (Biological Drug):ti,ab,kw OR (Drug, Biological):ti,ab,kw OR (Biologic Drugs):ti,ab,kw OR (Drugs, Biologic):ti,ab,kw OR (Biological Medicine):ti,ab,kw OR (Medicine, Biological):ti,ab,kw OR (Biological Medicines):ti,ab,kw OR (Medicines, Biological):ti,ab,kw OR (Biologic Medicines):ti,ab,kw OR (Medicines,Biologic):ti,ab,kw OR (Biologic Pharmaceuticals):ti,ab,kw OR (Pharmaceuticals, Biologic):ti,ab,kw OR (Biologic Drug):ti,ab,kw OR (Drug, Biologic):ti,ab,kw OR (Biological Drugs):ti,ab,kw OR (Drugs, Biological):ti,ab,kw OR (Natural Products):ti,ab,kw OR (Natural Product):ti,ab,kw OR (Product, Natural):ti,ab,kw
#8	MeSH descriptor: [Menopause] explode all trees
#9	MeSH descriptor: [Perimenopause] explode all trees
#10	(climacteric):ti,ab,kw OR (Change of Life, Female):ti,ab,kw OR (Climacterics):ti,ab,kw OR (Change of Life):ti,ab,kw OR (Life Change):ti,ab,kw OR (Life Changes):ti,ab,kw OR (quality of life):ti,ab,kw OR (health related quality of life):ti,ab,kw OR (menopause):ti,ab,kw OR (kupperman):ti,ab,kw
#11	#1 OR #2
#12	#3 OR #4 OR #5 OR #6 OR #7
#13	#8 OR #9 OR #10
#14	#11 AND #12 AND #13

Table 4. EMBASE

Search steps	Search terms
#1	'breast tumor'/exp AND [humans]/lim AND [english]/lim AND [clinical study]/lim AND [embase]/lim
#2	'breast neoplasm':ab,ti OR 'neoplasm, breast':ab,ti OR 'neoplasms, breast':ab,ti OR 'breast tumors':ab,ti OR 'tumor, breast':ab,ti OR 'tumors, breast':ab,ti OR 'breast cancer':ab,ti OR 'cancer, breast':ab,ti OR 'malignant tumor of breast':ab,ti OR 'breast malignant tumor':ab,ti OR 'breast malignant tumors':ab,ti OR 'cancer of the breast':ab,ti OR 'cancer of breast':ab,ti OR 'malignant neoplasm of breast':ab,ti OR 'breast malignant neoplasm':ab,ti OR 'breast malignant neoplasms':ab,ti OR 'mammary cancer':ab,ti OR 'cancer, mammary':ab,ti OR 'cancers, mammary':ab,ti OR 'mammary cancers':ab,ti OR 'mammary carcinoma, human':ab,ti OR 'carcinoma, human mammary':ab,ti OR 'carcinomas, human mammary':ab,ti OR 'human mammary carcinomas':ab,ti OR 'mammary carcinomas, human':ab,ti OR 'human mammary carcinoma':ab,ti OR 'mammary neoplasms, human':ab,ti OR 'human mammary neoplasm':ab,ti OR 'human

	mammary neoplasms':ab,ti OR 'neoplasm, human mammary':ab,ti OR 'neoplasms, human mammary':ab,ti OR 'mammary neoplasm, human':ab,ti OR 'breast carcinoma':ab,ti OR 'breast carcinomas':ab,ti OR 'carcinoma, breast':ab,ti OR 'carcinomas, breast':ab,ti
#3	'chinese medicine'/exp OR 'herbaceous agent'/exp OR 'biological product'/exp
#4	traditional:ab,ti AND chinese:ab,ti AND medicine:ab,ti OR (traditional:ab,ti AND medicine,:ab,ti AND chinese:ab,ti) OR (zhong:ab,ti AND yi:ab,ti AND xue:ab,ti) OR (chinese:ab,ti AND traditional:ab,ti AND medicine:ab,ti) OR (chinese:ab,ti AND medicine,:ab,ti AND traditional:ab,ti) OR (chinese:ab,ti AND drugs,:ab,ti AND plant:ab,ti) OR (chinese:ab,ti AND herbal:ab,ti AND drugs:ab,ti) OR (herbal:ab,ti AND drugs,:ab,ti AND chinese:ab,ti) OR (plant:ab,ti AND extracts,:ab,ti AND chinese:ab,ti) OR (chinese:ab,ti AND plant:ab,ti AND extracts:ab,ti) OR (extracts,:ab,ti AND chinese:ab,ti AND plant:ab,ti) OR (herbal:ab,ti AND medicine:ab,ti) OR (products,:ab,ti AND biological:ab,ti) OR (biological:ab,ti AND product:ab,ti) OR (product,:ab,ti AND biological:ab,ti) OR (biologic:ab,ti AND product:ab,ti) OR (product,:ab,ti AND biologic:ab,ti) OR (biologic:ab,ti AND products:ab,ti) OR biological:ab,ti OR biologic:ab,ti OR (biological:ab,ti AND drug:ab,ti) OR (drug,:ab,ti AND biological:ab,ti) OR (biologic:ab,ti AND drugs:ab,ti) OR (drugs,:ab,ti AND biologic:ab,ti) OR (biological:ab,ti AND medicine:ab,ti) OR (medicine,:ab,ti AND biological:ab,ti) OR (biological:ab,ti AND medicines:ab,ti) OR (medicines,:ab,ti AND biological:ab,ti) OR (biologic:ab,ti AND medicines:ab,ti) OR medicines,biologic:ab,ti OR (biologic:ab,ti AND pharmaceuticals:ab,ti) OR (pharmaceuticals,:ab,ti AND biologic:ab,ti) OR (biologic:ab,ti AND drug:ab,ti) OR (drug,:ab,ti AND biologic:ab,ti) OR (biological:ab,ti AND drugs:ab,ti) OR (drugs,:ab,ti AND biological:ab,ti) OR (natural:ab,ti AND products:ab,ti) OR (natural:ab,ti AND product:ab,ti) OR (product,:ab,ti AND natural:ab,ti)
#5	'menopause'/exp OR 'climacterium'/exp
#6	'climacterium':ab,ti OR 'menopause':ab,ti OR 'kupperman index':ab,ti
#7	#1 OR #2
#8	#3 OR #4
#9	#5 OR #6
#10	#7 AND #8 AND #9

Table 5. OVID

Search steps	Search terms
#1	Breast Neoplasms.mp.
#2	limit 1 to (english language and full text and humans)
#3	(Breast Neoplasm or Neoplasm, Breast or Neoplasms, Breast or Breast Tumors or Breast Tumor or Tumor, Breast or Tumors, Breast or Breast Cancer or Cancer, Breast or Malignant Tumor of Breast or Breast Malignant Tumor or Breast Malignant Tumors or Cancer of the Breast or Cancer of Breast or Malignant Neoplasm of Breast or Breast Malignant Neoplasm or Breast Malignant Neoplasms or Mammary Cancer or Cancer, Mammary or Cancers, Mammary or Mammary Cancers or Mammary Carcinoma,

	Human or Carcinoma, Human Mammary or Carcinomas, Human Mammary or Human Mammary Carcinomas or Mammary Carcinomas, Human or Human Mammary Carcinoma or Mammary Neoplasms, Human or Human Mammary Neoplasm or Human Mammary Neoplasms or Neoplasm, Human Mammary or Neoplasms, Human Mammary or Mammary Neoplasm, Human or Breast Carcinoma or Breast Carcinomas or Carcinoma, Breast or Carcinomas, Breast).mp. [mp=title, book title, abstract, original title, name of substance word, subject heading word, floating sub-heading word, keyword heading word, organism supplementary concept word, protocol supplementary concept word, rare disease supplementary concept word, unique identifier, synonyms]
#4	(Medicine, Chinese Traditional or Drugs, Chinese Herbal or Herbal Medicine or Biological Products).mp.
#5	limit 4 to (english language and full text and humans)
#6	(Traditional Chinese Medicine or Traditional Medicine, Chinese or Zhong Yi Xue or Chinese Traditional Medicine or Chinese Medicine, Traditional or Chinese Drugs, Plant or Chinese Herbal Drugs or Herbal Drugs, Chinese or Plant Extracts, Chinese or Chinese Plant Extracts or Extracts, Chinese Plant or Herbal Medicine or Products, Biological or Biological Product or Product, Biological or Biologic Product or Product, Biologic or Biologic Products or Biological or Biologic or Biological Drug or Drug, Biological or Biologic Drugs or Drugs, Biologic or Biological Medicine or Medicine, Biological or Biological Medicines or Medicines, Biological or Biologic Medicines or Medicines,Biologic or Biologic Pharmaceuticals or Pharmaceuticals, Biologic or Biologic Drug or Drug, Biologic or Biological Drugs or Drugs, Biological or Natural Products or Natural Product or Product, Natural).mp. [mp=title, book title, abstract, original title, name of substance word, subject heading word, floating sub-heading word, keyword heading word, organism supplementary concept word, protocol supplementary concept word, rare disease supplementary concept word, unique identifier, synonyms]
#7	Menopause.mp.
#8	limit 7 to (english language and full text and humans)
#9	Perimenopause.mp.
#10	limit 9 to (english language and full text and humans)
#11	(climacteric or menopause or kupperman).mp. [mp=title, book title, abstract, original title, name of substance word, subject heading word, floating sub-heading word, keyword heading word, organism supplementary concept word, protocol supplementary concept word, rare disease supplementary concept word, unique identifier, synonyms]
#12	limit 11 to (english language and full text and humans)
#13	2 or 3
#14	5 or 6
#15	8 or 10 or 12
#16	13 and 14 and 15

Table 6. Chinese National Knowledge Infrastructure

Search steps	Search terms
#1	TKA=('乳腺癌')('breast cancer') AND TKA=('更年期'+'绝经期'+'绝经综合'+'kupperman 评分'+'围绝经期综合征'+'更年期综合征'+'绝经前后诸证'+'经断前后诸证'+'绝经相关症状')('menopause'+'menopause syndrome'+'kupperman score'+'perimenopause syndrome'+'menopause syndrome'+'premenopausal and postmenopausal syndromes' +'menopausal related symptoms') AND TKA='中医药'+'中医'+'中药'+'传统医学'+'中草药'+'草药'+'药用植物'+'汤药'+'中药注射剂'+中成药'+'植物提取物') ('traditional Chinese medicine'+'traditional Chinese medicine'+'traditional Chinese medicine' + 'herbal medicine'+'medicinal plants'+'decoction drugs'+'traditional Chinese medicine injections'+'traditional Chinese patent medicines and simple preparations'+' plant extracts') AND TKA=('临床试验'+'临床研究'+'临床观察'+'随机'+'试验'+'分组'+'疗效')('Clinical trials'+'Clinical studies'+'Clinical observations'+'Randomization' + 'Trials' + 'Grouping'+'Efficacy')

Table 7. Wanfang database

Search steps	Search terms
#1	主题:(‘乳腺癌’)Theme:('breast cancer') and 主题:('更年期' or '绝经期' or '绝经综合征' or 'kupperman 评分' or '围绝经期综合征' or '更年期综合征' or '绝经前后诸证 'or‘经断前后诸证') and Theme:('menopause' or 'menopause syndrome' or 'kupperman score' or 'perimenopausal syndrome' or 'climacteric syndrome' or 'premenopausal and postmenopausal syndromes')and 主题:('中医药' or '中医' or '中药' or '传统医学' or '中草药' or '草药' or '药用植物' or '汤药' or '中药注射剂' or '中成药' or ' 植物提取物') and Theme:('traditional Chinese medicine' or 'Chinese herbal medicine' or 'herbal medicine' or 'medicinal plants' or' decoction drugs' or 'Chinese medicine injections' or' traditional Chinese patent medicines and simple preparations' or 'plant extracts') and 主题:('临床试验' or '临床研究' or '临床观察' or '随机' or '试验' or '分组' and Theme:('Clinical trials' or 'Clinical studies' or' Clinical observations' or 'Randomization' or 'Trials' or' Grouping ')

Table 8. Chinese Scientific Journals Database

Search steps	Search terms
#1	M=(乳腺癌)M=(breast cancer) AND M=(更年期 OR 绝经期 OR 绝经综合征 OR kupperman评分 OR 围绝 经期综合征 OR 更年期综合征 OR 绝经前后诸证 OR 经断前后诸证 OR 绝经相关症状) M=(menopause OR menopausal syndrome OR kupperman score OR perimenopausal syndrome OR menopausal syndrome OR pre and post menopausal syndrome OR menopausal symptoms) AND M=(中医药 OR 中医 OR 中药 OR 传统医学 OR 中草药 OR 草药 OR 药用植物 OR 汤药 OR 中药注射剂 OR 中成药 OR 植物提取物) M=(traditional Chinese medicine OR herbal medicine OR medicinal plant OR decoction OR traditional Chinese medicine injection OR traditional Chinese patent medicines and simple preparations OR plant extract) AND M=(临床试验 OR 临床研究 OR 临床观察 OR 随机 OR 试验 OR 分组 OR 疗效) M= (Clinical trials OR Clinical studies OR Clinical observations OR Randomized OR trials OR Grouping OR Efficacy)
#2	R=(乳腺癌) R=(breast cancer) AND R=(更年期 OR 绝经期 OR 绝经综合征 OR kupperman 评分 OR 围绝 经期综合征 OR 更年期综合征 OR 绝经前后诸证 OR 经断前后诸证 OR

	绝经相关症状) R=(menopause OR menopausal syndrome OR kupperman score OR perimenopausal syndrome OR menopausal syndrome OR pre and post menopausal syndrome OR menopausal symptoms) AND R=(中医药 OR 中医 OR 中药 OR 传统医学 OR 中草药 OR 草药 OR 药用植物 OR 汤药 OR 中药注射剂 OR 中成药 OR 植物提取物) R=(traditional Chinese medicine OR herbal medicine OR medicinal plant OR decoction OR traditional Chinese medicine injection OR traditional Chinese patent medicines and simple preparations OR plant extract) AND R=(临床试验 OR 临床研究 OR 临床观察 OR 随机 OR 试验 OR 分组 OR 疗效) R= (Clinical trials OR Clinical studies OR Clinical observations OR Randomized OR trials OR Grouping OR Efficacy)
#3	#1 OR #2
