# Supplementary material for: Efficacy and safety of traditional Chinese medicine in the treatment of menopause-like syndrome for breast cancer survivors: a systematic review and meta-analysis
Source: BMC Cancer. 2024 Jan 8;24:42. doi: 10.1186/s12885-023-11789-z (PMC10773128; doi:10.1186/s12885-023-11789-z)
Supplement: Supplementary file 3 — Additional file 3. Summary of all the included trials. [file 12885_2023_11789_MOESM3_ESM.pdf]

Summary of all of the included trails

Study	Formulation	Source	Species, concentration	Quality control reported? (Y/N)	Chemical analysis reported? (Y/N)
Bailing Shi,2010;	Traditional Chinese Medicine (TCM)	First Affiliated Hospital of Guangzhou University of Traditional Chinese Medicine	• Fruits of *Ligustrum lucidum Ait. (Nvzhenzi)*, 15g<br>• Dried herbs of *Eclipta prostrate L. (Mohanlian)*, 15g<br>• Fruits of Cornus officinalis Sieb.et Zucc. *(Shanyurou)*, 15g<br>• Dried roots and rhizomes of *Fallopia multiflora (Thunb.) Harald. (Heshouwu), 30g*<br>• Dried leaves of *Epimedium brevicornu Maxim. (Yinyanghuo)*, 10g<br>• Dried roots of *Curculigo orchioides Gaertn. (Xianmao), 10g*<br>• Dried roots of *Dioscorea opposita Thunb. (Shanyao), 24g*<br>• Dried roots of *Atractylodes macrocephala Koidz. (Baizhu), 15g*<br>• Dried sclerotia of *Poria cocos (Schw.) Wolf. (Fuling)*, 24g<br>• Fruits of *Ziziphus jujuba Mill. [Rhamnaceae] (Dazao), 15g*<br>• Dried roots of *Paeonia suffruticosa Andr. (Danpi), 15g*<br>• Dried peels of *Citrus reticulata Blanco (Chenpi)*, 6g<br>• Dried roots and rhizomes of *Glycyrrhiza uralensis Fisch (Gancao)*, 5g	N	N
Chenlu Liang et al, 2018;	Remifemin	Schaper & Brümmer GmbH & Co.KG	• Dried roots of *Cimicifuga heracleifolia Kom. (Shengma)*, 2g	N	Y
Chen Nie, 2018;	Yiqiyangyinjiedu(YQYYJD) formula	-	• Dried roots and rhizomes of *Codonopsis pilosula (Franch.) Nannf. (Dangshen), 20g*<br>• Dried roots of *Astragalus mongholicus Bunge. (Huangqi)*, 20g<br>• Dried roots of *Atractylodes macrocephala Koidz. (Baizhu), 20g*<br>• Dried roots and rhizomes of *Polygonatum kingianum Coll.et Hemsl. (Huangjing), 10g*<br>• Dried sclerotia of *Poria cocos* (Schw.) *Wolf. (Fuling)*, 15g<br>• Dried roots and rhizomes of *Rehmannia glutinosa Libosch. (Dihuang), 15g*<br>• Dried roots of *Dioscorea opposita Thunb. (Shanyao), 20g*<br>• Fruits of *Lycium barbarum L. (Gouqi), 15g*<br>• Dried seeds of *Psoralea corylifolia L. (Buguzhi)*, 15g<br>• Dried roots of *Curcuma phaeocaulis Val. (E'zhu), 10g*<br>• Dried roots of *Actinidia chinensis Planch. var. hispida C.F.Liang. (Tengligen), 15g*<br>• Dried herbs of *Hedyotis diffusa Willd. (Baihuasheshecao), 15g*	N	N
Chen Sun, 2009;	Xiaoyaoerxian(XYEX) Decoction	-	• Dried roots of *Curculigo orchioides Gaertn. (Xianmao), 9g*<br>• Dried roots and rhizomes of *Rehmannia glutinosa Libosch. (Dihuang), 15g*<br>• Dried leaves of *Epimedium brevicornu Maxim. (Yinyanghuo)*, 12g	N	N

			• Dried roots of *Bupleurum chinense DC.* *(Chaihu)*, 15g<br>• Fruits of *Citrus × aurantium L.* *(Zhiqiao)*, 12g<br>• Dried roots of *Angelica sinensis (Oliv.) Diels* *(Danggui)*, 12g<br>• Fruits of *Lycium barbarum L.* *(Gouqi)*, 15g<br>• Dried roots of *Paeonia lactiflora Pall.* *(Shaoyao), 12g*<br>• Dried sclerotia of *Poria cocos* (Schw.) *Wolf.* *(Fuling)*, 12g<br>• Dried roots of *Atractylodes macrocephala Koidz.* *(Baizhu), 12g*<br>• Dried roots and rhizomes of *Anemarrhena asphodeloides Bge.* *(Zhimu),* 9g<br>• Dried roots of *Salvia miltiorrhiza Bunge* *(Danshen), 12g*<br>• Dried herbs of *Hedyotis diffusa Willd.* *(Baihuasheshecao), 12g*<br>• Dried body of *Scolopendra subspinipes mutilans L. Koch* *(Wugong), 2*<br>• Dried roots and rhizomes of *Glycyrrhiza uralensis Fisch* *(Gancao)*, 6g		
Chen Wang et al,2019	Remifemin	Schaper & Brümmer GmbH & Co.KG	• Dried roots of *Cimicifuga heracleifolia Kom.* *(Shengma),* 2g	N	Y
Cheri L. Van Patten et al,2002	Soy	-	• soy beverage *(Dadou)*, 250 mL	N	N
Chundi Gao, 2019;	Erxian(EX) Decoction	Weifang Hospital of Traditional Chinese Medicine	• Dried roots of *Curculigo orchioides Gaertn.* *(Xianmao), 15g*<br>• Dried leaves of *Epimedium brevicornu Maxim.* *(Yinyanghuo)*, 20g<br>• Dried roots of *Morinda officinalis How* *(Bajitian),* 10g<br>• Dried peels of *Phellodendron amurense Rupr.* *(Huangbai),* 10g<br>• Dried roots and rhizomes of *Anemarrhena asphodeloides Bge.* *(Zhimu)*, 12g	N	N
Congshan Li et al., 2021	Chaihujialonggumuli(CHJLGML) Decoction		• Dried roots of *Angelica sinensis (Oliv.) Diels* *(Danggui),* 10g<br>• Dried peels of *Cinnamomum cassia Presl* *(Guizhi), 9g*<br>• Dried sclerotia of *Poria cocos* (Schw.) *Wolf.* *(Fuling)*, 12g<br>• Dried roots of *Pinellia ternata (Thunb.) Makino* *(Banxia), 9g*<br>• Dried herbs of *Scutellaria barbata D.Don* *(Banzhilian), 9g*<br>• Dried block of *Ostrea gigas Thunberg* *(Muli),* 30g<br>• Dried roots and rhizomes of *Codonopsis pilosula (Franch.) Nannf.* *(Dangshen), 12g*<br>• Dried roots and rhizomes of *Rheum officinale Baill.* *(Dahuang), 6g*<br>• Dried block of *FossiliaOssiaMastodi* *(Longgu),* 30 g<br>• Fruits of *Psoralea corylifolia Linn.* *(Buguzhi),* 12 g<br>• Fruits of *Lycium barbarum L.* *(Gouqi),* 12 g<br>• Dried seeds of *Cuscuta chinensis Lam.* *(Tusizi),* 12 g<br>• Dried leaves of *Epimedium brevicornu Maxim.* *(Yinyanghuo)*, 12g<br>• Dried roots of *Radix Dipsaci Asperoidis* *(Xuduan)*, 15g	N	N
Guilan Tan, 2014;	TAM	Guangzhou University of Traditional Chinese Medicine	•Dried leaves of *Taxillus sutchuenensis (Lecomte) Danser* *(Sangjisheng), 30g*<br>• Dried leaves of *Epimedium brevicornu Maxim.* *(Yinyanghuo)*, 15g<br>• Dried seeds of *Cuscuta chinensis Lam.* *(Tusizi),* 15g	N	N
Haisong Lu et	Xiaogeng(XG	-	• Dried roots of *Radix Dipsaci Asperoidis* *(Xuduan)*, 10g	N	N

al, 2016	) powder		• Dried roots and rhizomes of *Fallopia multiflora (Thunb.) Harald. (Heshouwu), 10g*<br>• Fruits of *Lycium barbarum L. (Gouqi)*, 15g<br>• Fruits of *Ligustrum lucidum Ait. (Nvzhenzi)*, 10g<br>• Dried roots and rhizomes of *Rehmannia glutinosa Libosch. (Dihuang), 15g*<br>• Dried roots and rhizomes of *Anemarrhena asphodeloides Bge (Zhimu)*. 10g<br>• Dried roots of *Angelica sinensis (Oliv.) Diels (Danggui)*, 10g<br>• Dried roots of *Paeonia lactiflora Pall. (Shaoyao)*, 10g<br>• Dried block of *FossiliaOssiaMastodi (Longgu)*, 30g<br>• Dried block of *Ostrea gigas Thunberg (Muli)*, 30g<br>• Dried roots and rhizomes of *Glycyrrhiza uralensis Fisch (Gancao)*, 6g<br>• Dried stems of *Uncariarhynchophylla(Miq.)Miq. ex Havil. (Gouteng), 10g*<br>• Dried roots of *CyathulaofficinalisKuan. (Chuanniuxi), 10g*<br>• Dried roots of *Curcuma wenyujin Y. H. Chen et C. Ling. (Yujin), 10g*<br>• Dried stems of *Cauls Polugoni Multiflori. (Yejiaoteng), 30g*<br>• Dried body of *Bombyx Batryticatus. (Jiangcan), 10g*		
Han Xiao et al, 2019	Jiaweizhibaidihuang(JWZBDH) Decoction	Jiangyin Tianjiang Pharmaceutical Co., Ltd	• Dried roots and rhizomes of *Alisma plantago-aquatica subsp. orientale (Sam.) (Zexie), 10g*<br>• Dried roots and rhizomes of *Anemarrhena asphodeloides Bge (Zhimu)*. 10g<br>• Dried peels of *Phellodendron amurense Rupr. (Huangbai)*, 6g<br>• Dried roots and rhizomes of *Rehmannia glutinosa Libosch. (Dihuang), 20 g*<br>• Dried roots of *Dioscorea opposita Thunb. (Shanyao), 10g*<br>• Fruits of Cornus officinalis Sieb.et Zucc. *(Shanyurou)*, 12g<br>• Dried roots of *Paeonia suffruticosa Andr. (Danpi), 10g*<br>• Dried sclerotia of *Poria cocos* (Schw.) *Wolf. (Fuling)*, 10g<br>• Fruits of *Ligustrum lucidum Ait. (Nvzhenzi)*, 10g<br>• Dried leaves of *Epimedium brevicornu Maxim. (Yinyanghuo)*, 10g<br>• Dried herbs of *Eclipta prostrate L. (Mohanlian)*, 15g<br>• Dried roots and rhizomes of *Glycyrrhiza uralensis Fisch (Gancao)*, 5g	N	N
Hongxia Li et al., 2020	Qianyangfengsui(QYFS) pellet	-	• Dried peels of *Phellodendron amurense Rupr. (huangbai)*, 10g<br>• Dried roots and rhizomes of *Glycyrrhiza uralensis Fisch (Gancao)*, 6g<br>• Dried block of *FossiliaOssiaMastodi (Longgu)*, 30 g<br>• Dried block of *Ostrea gigas Thunberg (Muli)*, 30g<br>• Shell of *Chinemys reevesii (Zhiguiban), 20g*<br>• Fruits of *Hordeum vulgare L. (Fuxiaomai)*, 30g<br>•Dried leaves of *Taxillus sutchuenensis (Lecomte) Danser (Sangjisheng), 15g*<br>• Dried roots of *Aconitum carmichaeli Debx. (Wutou), 10g*	N	N
Huifen Yang	Erzhi(EZ) pill	-	• Dried herbs of *Eclipta prostrate L. (Mohanlian)*, 10g	N	N

et al, 2015	+Guizhi(GZ) Decoction		• Fruits of *Ligustrum lucidum Ait.* *(Nvzhenzi)*, 10g<br>• Dried roots of *Paeonia lactiflora Pall.* *(Shaoyao), 10g*<br>• Dried roots and rhizomes of *Glycyrrhiza uralensis Fisch (Gancao)*, 5g<br>• Dried peels of *Cinnamomum cassia Presl* *(Guizhi), 6g*		
Huifen Yang et al, 2016	Erzhi(EZ) pill +Guizhi(GZ) Decoction	-	• Fruits of *Ligustrum lucidum Ait.* *(Nvzhenzi)*, 10g<br>• Dried herbs of *Eclipta prostrate L.* *(Mohanlian)*, 10g<br>• Dried roots and rhizomes of *Glycyrrhiza uralensis Fisch (Gancao)*, 6g<br>• Dried peels of *Cinnamomum cassia Presl* *(Guizhi), 9g*<br>• Dried roots of *Paeonia lactiflora Pall.* *(Shaoyao), 9g*	N	Y
Hui Liu et al, 2016	Yiguan(YG) Decoction	The First Affiliated Hospital of Hunan University of Traditional Chinese Medicine	• Dried roots of *Curculigo orchioides Gaertn.* *(Xianmao), 15g*<br>• Dried leaves of *Epimedium brevicornu Maxim.* *(Yinyanghuo)*, 15g<br>• Dried peels of *Phellodendron amurense Rupr.* *(Huangbai)*, 9g<br>• Dried roots and rhizomes of *Anemarrhena asphodeloides Bge* *(Zhimu).* 9g<br>• Dried roots of *Angelica sinensis (Oliv.) Diels* *(Danggui)*, 9g<br>• Dried block of *Ostrea gigas Thunberg* *(Muli)*, 15g<br>• Fruits of *Hordeum vulgare L.* *(Fuxiaomai)*, 15g<br>• Dried roots of *Morinda officinalis How* *(Bajitian)*, 9g	N	N
Hui Zhang,2018	Erjialonggu(EJLG) Decoction	The First Affiliated Hospital of Anhui University of Traditional Chinese Medicine	• Dried block of *FossiliaOssiaMastodi* *(Longgu)*, 15g<br>• Dried block of *Ostrea gigas Thunberg* *(Muli)*, 15g<br>• Dried roots of *Paeonia lactiflora Pall.* *(Shaoyao)*, 25g<br>• Fruits of *Ligustrum lucidum Ait.* *(Nvzhenzi)*, 15g<br>• Fruits of *Ziziphus jujuba Mill. [Rhamnaceae]* *(Dazao), 25g*<br>• Dried roots and rhizomes of *Glycyrrhiza uralensis Fisch (Gancao)*, 5g<br>• Dried peels of *Cinnamomum cassia Presl* *(Guizhi), 6g*<br>• Dried roots of *Ophiopogon japonicus (L.f) Ker-Gawl* *(Maidong). 12g*<br>• Dried stems of *Bambusa tuldoides Munro.* *(Zhuru), 10g*<br>• Dried roots of *Curcuma wenyujin Y. H. Chen et C. Ling.* *(Yujin), 10g*<br>• Dried stems of *Coptis chinensis Franch.* *(Huanglian), 3g*	N	N
Jingru Song, 2019	Chaihujialonggumuli(CHJLGML) Decoction	The Fourth Clinical Medical College of Xinjiang Medical University	• Dried roots of *Bupleurum chinense DC.* *(Chaihu), 10g*<br>• Dried herbs of *Scutellaria barbata D.Don* *(Banzhilian), 10g*<br>• Dried block of *FossiliaOssiaMastodi* *(Longgu)*, 30 g<br>• Dried block of *Ostrea gigas Thunberg* *(Muli)*, 30g<br>• Dried roots and rhizomes of *Rehmannia glutinosa Libosch.* *(Dihuang), 20g*<br>• Dried roots of *Salvia miltiorrhiza Bunge* *(Danshen), 15g*<br>• Dried roots and rhizomes of *Codonopsis pilosula (Franch.) Nannf.* *(Dangshen), 12g*<br>• Dried roots and rhizomes of *Rheum officinale Baill.* *(Dahuang), 3g*<br>• Dried sclerotia of *Poria cocos* (Schw.) *Wolf. (Fuling)*, 12g<br>• Dried peels of *Cinnamomum cassia Presl* *(Guizhi), 6g*<br>• Dried roots of *Pinellia ternata (Thunb.) Makino* *(Banxia), 12g*<br>• Fruits of *Hordeum vulgare L.* *(Fuxiaomai)*, 15g<br>• Fruits of *Liliumbrowniivar.viridulum* *(Baihe), 12g*	N	N

Juan Zhou,2020	Xiao yao an kun(XYAK) Decoction		• Dried roots of *Bupleurum chinense DC.* (Caihu), 8g<br>• Dried roots of *Atractylodes macrocephala Koidz.* (Baizhu), 30g<br>• Dried sclerotia of *Poria cocos* (Schw.) *Wolf.* (Fuling), 15g<br>• Dried roots of *Angelica sinensis (Oliv.) Diels* (Danggui), 15g<br>• Dried roots of *Paeonia lactiflora Pall.* (Shaoyao), 15g<br>• Fruits of *Ligustrum lucidum Ait.* (Nvzhenzi), 15g<br>• Dried herbs of *Eclipta prostrate L.* (Mohanlian), 15g<br>• Dried roots and rhizomes of *Rehmannia glutinosa Libosch.* (Dihuang), 15g<br>• Fruits of Cornus officinalis Sieb.et Zucc. (Shanyurou), 15g<br>• Dried peels of *Fructus Citri Reticulatae Immaturus.* (Qingpi), 10g<br>• Dried herbs of *Hedyotis diffusa Willd.* (Baihuasheshecao), 15g<br>• Dried roots and rhizomes of *Glycyrrhiza uralensis Fisch* (Gancao), 10g<br>• Dried seeds of *TangerineSeed.* (Jvhe), 15g<br>• Dried seeds of *Litchi chinensis Sonn.* (Lizhihe), 15g<br>• Dried stems of (Pugongying), 15g<br>• Dried body of *Gekko japonicus Dumeril et Bibron.* (Bihu), 10g	N	N
Judith S. Jacobson et al,2001	Black Cohosh		• Fruits of *Cimicifuga heracleifolia Kom.* (Shengma)	N	N
Junwen Pei et al, 2019	Danzhixiaoyao(DZXY) powder+Erxian(EX) Decoction	ChinaResourcesSanjiu Medical&Pharmaceutical Co.,Ltd.	• Dried roots of *Paeonia suffruticosa Andr.* (Danpi), 9g<br>• Dried roots of *Bupleurum chinense DC.* (Chaihu), 10 g<br>• Dried roots of *Angelica sinensis (Oliv.) Diels* (Danggui), 15 g<br>• Dried peels of *Phellodendron amurense Rupr.* (Huangbai), 15g<br>• Dried roots of *Atractylodes macrocephala Koidz.* (Baizhu), 30g<br>• Dried sclerotia of *Poria cocos* (Schw.) *Wolf.* (Fuling), 30g<br>• Dried roots of *Curculigo orchioides Gaertn.* (Xianmao), 15g<br>• Dried leaves of *Epimedium brevicornu Maxim.* (Yinyanghuo), 15g<br>• Dried roots and rhizomes of *Glycyrrhiza uralensis Fisch* (Gancao), 6g<br>• Dried roots of *Paeonia lactiflora Pall.* (Shaoyao), 15g<br>• Dried roots and rhizomes of *Anemarrhena asphodeloides Bge* (Zhimu). 15g<br>• Dried roots of *Morinda officinalis How* (Bajitian), 15g	N	N
Junyuan Cai et al., 2021	Fuzhengxiaoliu(FZXL) Decoction		• Dried stems of *Cremastra appendiculata (D.Don) Makino* (Shancigu), 15g<br>• Dried roots of *Sparganium stoloniferum Buch.-Ham.* (Sanling), 10g<br>• Dried herbs of *Agrimonia pilosa Ledeb.* (Xianhecao),15g<br>• Fruits of *Liliumbrowniivar.viridulum* (Baihe), 10g<br>• Fruits of *Coix lacryma-jobi L.* (Yiyiren), 15g<br>• Dried roots of *Polyporus umbellatus (Pers.) Fries.* (Zhuling), 10g<br>• Dried roots of *Pinellia ternata (Thunb.) Makino* (Banxia), 10g<br>• Dried herbs of *Hedyotis diffusa Willd.* (Baihuasheshecao), 30 g<br>• Dried roots of *Curcuma phaeocaulis Val.* (E’zhu), 15g<br>• Dried peels of *Citrus reticulata Blanco* (Chenpi), 10g	N	N

Kaili Xu, 2019	Dangguiliuhuang(DGLH) Decoction	Affiliated Hospital of Shandong University of Traditional Chinese Medicine	• Dried roots and rhizomes of *Glycyrrhiza uralensis Fisch (Gancao)*, 10g<br>• Dried roots of *Angelica sinensis (Oliv.) Diels (Danggui), 30*g<br>• Dried roots and rhizomes of *Rehmannia glutinosa Libosch. (Dihuang), 40g*<br>• Dried roots and rhizomes of *Anemarrhena asphodeloides Bge (Zhimu).* 15g<br>• Dried peels of *Phellodendron amurense Rupr. (Huangbai),* 15g<br>• Dried roots of *Astragalus mongholicus Bunge. (Huangqi)*, 20g	N	N
Lan Luo et al, 2019	TAM	Yangzijiang Pharmaceutical Group Co., Ltd	• Dried roots and rhizomes of *Codonopsis pilosula (Franch.) Nannf. (Dangshen), 20g*<br>• Dried sclerotia of *Poria cocos* (Schw.) *Wolf. (Fuling)*, 15g<br>• Fruits of *Ligustrum lucidum Ait. (Nvzhenzi)*, 15g<br>• Fruits of Cornus officinalis Sieb.et Zucc. *(Shanyurou)*, 15g<br>• Dried roots of *Bupleurum chinense DC. (Chaihu), 15g*<br>• Dried roots of *Paeonia lactiflora Pall. (Shaoyao), 15g*<br>• Dried roots of *Curcuma wenyujin Y. H. Chen et C. Ling. (Yujin), 15g*<br>• Fruits of *Akebia trifoliata(Thunb)Koidz var. (Mutong), 15*g	N	N
Limin Zhu,2020	Ruyanning(RYN) formula		• Dried roots and rhizomes of *Rehmannia glutinosa Libosch. (Dihuang)*<br>• Fruits of Cornus officinalis Sieb.et Zucc. *(Shanyurou)*,<br>• Dried roots and rhizomes of *Anemarrhena asphodeloides Bge (Zhimu).*<br>• Dried peels of *Phellodendron amurense Rupr (Huangbai).*<br>• Dried roots of *Rumex hymenosepalus. (Hongshen)*<br>• Dried roots of *Astragalus mongholicus Bunge. (Huangqi).*<br>• Fruits of *Akebia trifoliata(Thunb)Koidz var. (Mutong)*	N	N
Lina Sheng, 2014	Yangshenshugan(YSSG) Decoction		• Dried roots and rhizomes of *Rehmannia glutinosa Libosch. (Dihuang), 15g*<br>• Fruits of Cornus officinalis Sieb.et Zucc. *(Shanyurou)*, 15g<br>• Dried roots of *Dioscorea opposita Thunb. (Shanyao), 20g*<br>• Dried peels of *Phellodendron amurense Rupr. (Huangbai),* 12g<br>• Dried stems of *Bambusa tuldoides Munro. (Zhuru),12g*<br>• Fruits of *Schisandra chinensis (Turcz.) Baill. (Wuweizi), 12g*<br>• Dried roots of *Curcuma wenyujin Y. H. Chen et C. Ling (Yujin), 12g*<br>• Dried roots and rhizomes of *Alisma plantago-aquatica subsp. orientale (Sam.) (Zexie), 15g*<br>• Dried roots of *Paeonia suffruticosa Andr. (Danpi), 15g*<br>• Dried sclerotia of *Poria cocos* (Schw.) *Wolf. (Fuling)*, 15g<br>• Dried roots and rhizomes of *Anemarrhena asphodeloides Bge (Zhimu).* 12g<br>• Dried roots of *Bupleurum chinense DC. (Chaihu), 12g*<br>• Dried roots and rhizomes of *Glycyrrhiza uralensis Fisch (Gancao)*, 6g	N	N
Liujing Ou, 2018	TAM		• Dried roots and rhizomes of *Rehmannia glutinosa Libosch. (Dihuang), 20g*<br>• Fruits of *Psoralea corylifolia Linn. (Buguzhi),* 15g<br>• Dried seeds of *Cuscuta chinensis Lam. (Tusizi),* 15g	N	N

Ming Feng et al,2021	Sanhuang(SH) Decoction	Jiangsu Province Hospital of TCM (Nanjing, China).	• Dried roots of *Angelica sinensis (Oliv.) Diels (Danggui)*, 15g<br>• Fruits of *Lycium barbarum L. (Gouqi), 15g*<br>• Fruits of Cornus officinalis Sieb.et Zucc. *(Shanyurou)*, 10g<br>• Dried roots and rhizomes of *Ligusticum chuanxiong Hort. (Chuanxiong)*, 10g<br>• Dried roots and rhizomes of *Rheum officinale Baill. (Dahuang), 10g*<br>• Dried roots of *Curcuma phaeocaulis Val. (E'zhu), 30g*<br>• Dried roots of *Astragalus mongholicus Bunge. (Huangqi)*, 10g<br>• Dried roots of *Bupleurum chinense DC. (Chaihu), 10g*<br>• Dried block of *FossiliaOssiaMastodi (Longgu)*, 30 g<br>• Dried block of *Ostrea gigas Thunberg (Muli)*, 30g	N	N
Rongfei Jiang, 2021	Chaihujialonggumuli(CHJLGML) Decoction	The First Affiliated Hospital of Tianjin University of Traditional Chinese Medicine	• Dried roots and rhizomes of *Codonopsis pilosula (Franch.) Nannf. (Dangshen), 15g*<br>• Dried sclerotia of *Poria cocos* (Schw.) *Wolf. (Fuling)*, 10g<br>• Dried herbs of *Scutellaria barbata D.Don (Banzhilian), 10g*<br>• Dried roots and rhizomes of *Rheum officinale Baill. (Dahuang), 5g*<br>• Dried peels of *Cinnamomum cassia Presl (Guizhi), 10g*<br>• Dried roots of *Pinellia ternata (Thunb.) Makino (Banxia), 10g*	N	N
Rui Qiang, 2020	Banxiaxiexin(BXXX) Decoction		• Dried herbs of *Scutellaria barbata D.Don (Banzhilian), 9g*<br>• Dried roots and rhizomes of *Codonopsis pilosula (Franch.) Nannf. (Dangshen), 15g*<br>• Dried roots and rhizomes of *Anemarrhena asphodeloides Bge (Zhimu).* 10g<br>• Dried roots and rhizomes of *Glycyrrhiza uralensis Fisch (Gancao)*, 6g<br>• Dried peels of *Cinnamomum cassia Presl (Guizhi), 3g*<br>• Dried roots of *Pinellia ternata (Thunb.) Makino (Banxia), 9g*<br>• Dried roots of *Astragalus mongholicus Bunge. (Huangqi)*, 9g<br>• Fruits of *Hordeum vulgare L. (Fuxiaomai)*, 30g<br>• Fruits of *Liliumbrowniivar.viridulum (Baihe), 20g*	N	N
Ruiqing Sui, 2019	Zishuihanmu(ZSHM) formula		• Dried roots and rhizomes of *Rehmannia glutinosa Libosch. (Dihuang), 12g*<br>• Fruits of *Lycium barbarum L. (Gouqi), 10g*<br>• Fruits of Cornus officinalis Sieb.et Zucc. *(Shanyurou)*, 10g<br>• Dried roots of *Dioscorea opposita Thunb. (Shanyao), 15g*<br>• Dried sclerotia of *Poria cocos* (Schw.) *Wolf. (Fuling)*, 10g<br>• Some powder of *Galli Gigerii Endothelium Corneum (Jineijin), 15g*<br>• Dried roots of *Paeonia lactiflora Pall. (Shaoyao), 10g*<br>• Fruits of *Hordeum vulgare L. (Fuxiaomai)*, 15g	N	N
Suzhen Lin et al, 2016	Yiguan(YG) Decoction		• Dried roots of *Angelica sinensis (Oliv.) Diels (Danggui)*, 9g<br>• Dried roots and rhizomes of *Rehmannia glutinosa Libosch. (Dihuang), 15g*<br>• Dried roots of *Ophiopogon japonicus (L.f) Ker-Gawl (Maidong). 15g*<br>• Dried roots of *Glehnia littoralis Fr. Schmidtex Miq. (Beishashen), 15g*<br>• Fruits of *Lycium barbarum L. (Gouqi), 15g*	N	N

Ting Li et al., 2020	Xiaoyaoankun(XYAK) Decoction	• Dried roots of *Bupleurum chinense DC. (Chaihu)*<br>• Dried roots of *Atractylodes macrocephala Koidz. (Baizhu)*<br>• Dried sclerotia of *Poria cocos* (Schw.) *Wolf. (Fuling)*.<br>• Dried roots of *Angelica sinensis (Oliv.) Diels. (Danggui)*<br>• Dried roots of *Paeonia lactiflora Pall. (Shaoyao)*<br>• Fruits of *Ligustrum lucidum Ait (Nvzhenzi)*.<br>• Dried herbs of *Eclipta prostrate L. (Mohanlian)*<br>• Dried roots and rhizomes of *Rehmannia glutinosa Libosch. (Dihuang)*<br>• Fruits of Cornus officinalis Sieb.et Zucc. *(Shanyurou)*,<br>• Dried peels of *Fructus Citri Reticulatae Immaturus (Qingpi)*<br>• Dried herbs of *Hedyotis diffusa Willd. (Baihuasheshecao)*<br>• Dried roots and rhizomes of *Glycyrrhiza uralensis Fisch (Gancao)*<br>• Dried seeds of *TangerineSeed (Juhe)*<br>• Dried seeds of *Litchi chinensis Sonn. (Lizhihe)*<br>• Dried body of *Gekko japonicus Dumeril et Bibron. (Bihu)*<br>• Dried stems of *Taraxacum mongolicum Hand. -Mazz. (Pugongying)*	N	N
Weikang Zhu,2020	TAM	• Dried roots and rhizomes of *Rehmannia glutinosa Libosch. (Dihuang), 20g*<br>• Dried seeds of *Cuscuta chinensis Lam. (Tusizi)*, 15g<br>• Dried roots of *Bupleurum chinense DC. (Chaihu), 15g*<br>• Dried herbs of *Scutellaria barbata D.Don (Banzhilian), 30g*<br>• Dried roots and rhizomes of *Codonopsis pilosula (Franch.) Nannf. (Dangshen), 15g*<br>• Dried roots and rhizomes of *Glycyrrhiza uralensis Fisch (Gancao)*, 9g<br>• Dried roots of *Pinellia ternata (Thunb.) Makino (Banxia), 9g*<br>• Dried roots and rhizomes of *Alisma plantago-aquatica subsp. orientale (Sam.) (Zexie), 15g*	N	N
Xiaoling Wang, 2018	TAM	• Dried roots and rhizomes of *Rehmannia glutinosa Libosch. (Dihuang), 20g*<br>• Dried herbs of *Eclipta prostrate L. (Mohanlian)*, 20g<br>• Fruits of *Ligustrum lucidum Ait. (Nvzhenzi)*, 20g<br>• Dried peels of *Cinnamomum cassia Presl (Guizhi), 15g*<br>• Dried roots of *Paeonia lactiflora Pall. (Shaoyao), 15g*<br>• Dried roots and rhizomes of *Glycyrrhiza uralensis Fisch (Gancao)*, 10g	N	N
Xiaomei Wu et al, 2021	TAM	• Dried roots and rhizomes of *Rehmannia glutinosa Libosch. (Dihuang), 30g*<br>• Dried stems of *Taraxacum mongolicum Hand. -Mazz. (Pugongying), 30g*<br>• Fruits of *Euryale ferox Salisb. ex DC. (Qianshi), 30g*<br>• Fruits of *Liliumbrowniivar.viridulum (Baihe), 15g*<br>• Fruits of *Akebia trifoliata(Thunb)Koidz var. (Mutong),* 15g<br>• Dried roots of *Curcuma wenyujin Y. H. Chen et C. Ling. (Yujin), 15g*<br>• Fruits of *Hordeum vulgare L. (Fuxiaomai)*, 30g<br>• Dried body of *Scolopendra subspinipes mutilans L. Koch (Wugong), 3*	N	N

			• Fruits of *Hordeum vulgare L. (Fuxiaomai)*, 15g<br>• Dried roots of *Ophiopogon japonicus (L.f) Ker-Gawl (Maidong). 15g*<br>• Dried roots of *Paeonia lactiflora Pall. (Shaoyao), 15g*<br>• Dried roots of *Paeonia suffruticosa Andr. (Danpi), 10g*<br>• Fruits of Cornus officinalis Sieb.et Zucc. *(Shanyurou)*, 10g<br>• Dried leaves of *Epimedium brevicornu Maxim. (Yinyanghuo)*, 10g<br>• Dried roots of *Bupleurum chinense DC. (Chaihu),* 10 g<br>• Dried roots and rhizomes of *Glycyrrhiza uralensis Fisch (Gancao)*, 9g		
Xiaozhen Liang, 2011	Liuweidihuang(LWDH) Decoction		• Dried roots and rhizomes of *Rehmannia glutinosa Libosch. (Dihuang), 24g*<br>• Fruits of Cornus officinalis Sieb.et Zucc. *(Shanyurou)*, 12g<br>• Dried roots of *Dioscorea opposita Thunb. (Shanyao), 12g*<br>• Dried sclerotia of *Poria cocos* (Schw.) *Wolf. (Fuling)*, 9g<br>• Dried roots of *Paeonia suffruticosa Andr. (Danpi), 9g*<br>• Fruits of *Ligustrum lucidum Ait. (Nvzhenzi)*, 15g<br>• Dried herbs of *Eclipta prostrate L. (Mohanlian)*, 15g<br>• Dried block of *Ostrea gigas Thunberg (Muli)*, 30g<br>• Shell of *Chinemys reevesii (Zhiguiban), 30g*<br>• Dried roots and rhizomes of *Alisma plantago-aquatica subsp. orientale (Sam.) (Zexie), 9g*	N	N
Yang Fu, 2016	Heixiaoyao(HXY) Powder+Shensiwei(SSW) Decoction		• Dried roots of *Bupleurum chinense DC. (Chaihu), 15g*<br>• Dried roots of *Paeonia lactiflora Pall. (Shaoyao)*, 15g<br>• Dried roots of *Atractylodes macrocephala Koidz. (Baizhu), 12g*<br>• Dried roots and rhizomes of *Glycyrrhiza uralensis Fisch (Gancao)*, 10g<br>• Dried roots and rhizomes of *Rehmannia glutinosa Libosch. (Dihuang), 12g*<br>• Dried seeds of *Cuscuta chinensis Lam. (Tusizi),* 12 g<br>• Dried leaves of *Epimedium brevicornu Maxim. (Yinyanghuo)*, 12g<br>• Fruits of *Psoralea corylifolia Linn. (Buguzhi),* 12 g<br>• Dried roots of *Angelica sinensis (Oliv.) Diels (Danggui), 12*g<br>• Dried sclerotia of *Poria cocos* (Schw.) *Wolf. (Fuling)*, 20g<br>• Fruits of *Lycium barbarum L. (Gouqi), 12g*<br>• Dried stems of *Fallopia multiflora.(Yejiaoteng), 12g*	N	N
Yemei Li et al, 2022	Jiaweifangjidihuang(JWFJDH) Decoction	Wuyi Hospital of Traditional Chinese Medicine, Jiangmen City, Guangdong Province	• Dried roots and rhizomes of *Rehmannia glutinosa Libosch. (Dihuang), 30g*<br>• Fruits of *Liliumbrowniivar.viridulum (Baihe), 30g*<br>• Dried roots of *Stephania tetrandra S.Moore (Fangji),10g*<br>• Fruits of *Coix lacryma-jobi L.* (Yiyiren), 30g<br>• Dried peels of *Cinnamomum cassia Presl (Guizhi), 6g*<br>• Dried roots of *Pinellia ternata (Thunb.) Makino (Banxia), 30g*<br>• Dried roots and rhizomes of *Glycyrrhiza uralensis Fisch (Gancao)*, 10g<br>• Dried block of *FossiliaOssiaMastodi (Longgu),* 30 g<br>• Dried block of *Ostrea gigas Thunberg (Muli)*, 30g<br>• Dried roots and rhizomes of *Anemarrhena asphodeloides Bge (Zhimu).*	N	N

			10g<br>• Fruits of *Ziziphus jujuba Mill. [Rhamnaceae] (Dazao), 30g*<br>• Dried roots and rhizomes of *Fallopia multiflora (Thunb.) Harald. (Heshouwu), 30g*<br>• Dried sclerotia of *Poria cocos* (Schw.) *Wolf. (Fuling)*, 15g		
Yining Song et al, 2014	Remifemin	Schaper & Brümmer GmbH & Co.KG	• Dried roots of *Cimicifuga heracleifolia Kom. (Shengma)*, 2g	N	Y
Zhihui Tao et al, 2020	Yishenkangai (YSKA) formula		• Fruits of *Ligustrum lucidum Ait. (Nvzhenzi)*, 12g<br>• Dried herbs of *Eclipta prostrate L. (Mohanlian)*, 12g<br>• Dried block of *Ostrea gigas Thunberg (Muli)*, 15g<br>• Dried sclerotia of *Poria cocos* (Schw.) *Wolf. (Fuling)*, 15g<br>• Some powder of *Galli Gigerii Endothelium Corneum (Jineijin), 12g*<br>• Dried roots and rhizomes of *Polygonatum kingianum Coll.et Hemsl. (Huangjing), 30g*<br>• Fruits of *Hordeum vulgare L. (Fuxiaomai)*, 15g<br>• Dried stems of *Fallopia multiflora. (Yejiaoteng), 15g*<br>• Dried roots of *Atractylodes macrocephala Koidz. (Baizhu)*, 15g<br>•Dried leaves of *Taxillus sutchuenensis (Lecomte) Danser (Sangjisheng), 15g*<br>• Dried stems of *Cremastra appendiculata (D.Don) Makino (Shancigu), 12g*	N	N
Zhipei Han et al., 2021	Ruyanning(RYN) formula		• Dried roots and rhizomes of *Rehmannia glutinosa Libosch. (Dihuang),* 15~18g<br>• Fruits of Cornus officinalis Sieb.et Zucc. *(Shanyurou)*, 10~12g<br>• Dried roots and rhizomes of *Anemarrhena asphodeloides Bge (Zhimu).* 10~12g<br>• Dried roots and rhizomes of *Amorphophallus rivieri Durieu. (Sheliugu),* 25~30g<br>• Dried peels of *Phellodendron amurense Rupr. (Huangbai),* 10~12g<br>• Fruits of *Akebia trifoliata(Thunb)Koidz var. (Mutong), 10*~12g<br>• Dried herbs of *Hedyotis chrysotricha (Palib.) Merr. (Shijianchuan),* 12~15g<br>• Dried roots of *Astragalus mongholicus Bunge. (Huangqi)*, 25~30g<br>• Dried herbs of *Hedyotis diffusa Willd. (Baihuasheshecao),* 25~30g<br>• Dried roots of *Bupleurum chinense DC. (Chaihu),* 10~12g	N	N
Ziyi Yan, 2021	Qingxinzishen(QXZS) Decoction		• Fruits of *Ziziphus jujuba Mill. [Rhamnaceae] (Dazao), 15g*<br>• Dried roots and rhizomes of *Rehmannia glutinosa Libosch. (Dihuang), 15g*<br>• Fruits of *Ziziphus jujuba Mill. var. spinosa(Bunge)Hu ex H. F. Chou. (Suanzaoren), 15g*<br>• Dried roots of *Salvia miltiorrhiza Bunge. (Danshen), 10g*<br>• Dried fruits of *Cornus officinalis Sieb.et Zucc. (Shanyurou)*, 9g<br>• Dried roots of *Astragalus mongholicus Bunge. (Huangqi)*, 3g<br>• Dried stems of *Uncariarhynchophylla(Miq.)Miq. ex Havil. (Gouteng),*	N	N

16g

- Dried roots and rhizomes of *Pseudostellaria heterophylla (Miq.) Pax. (Taizishen), 10g*
- Fruits of *Hordeum vulgare L. (Fuxiaomai),* 30g
- Dried roots of *Curcuma wenyujin Y. H. Chen et C. Ling. (Yujin), 10g*
- Dried stems of *Spatholobus suberectus Dunn.(Jixueteng),10g*
- Fruits of *Nelumbo nucifera Gaertn. (Lianzixin),5g*
- Dried stems of *Solanum lyratum Thunb. (Baiying), 10g*
