# Supplementary material for: Efficacy and safety of traditional Chinese medicine in the treatment of menopause-like syndrome for breast cancer survivors: a systematic review and meta-analysis
Source: BMC Cancer. 2024 Jan 8;24:42. doi: 10.1186/s12885-023-11789-z (PMC10773128; doi:10.1186/s12885-023-11789-z)
Supplement: Supplementary file 4 — Additional file 4. Sensitivity analysis images. [file 12885_2023_11789_MOESM4_ESM.pdf]

Study or Subgroup	Experimental Mean	Experimental SD	Experimental Total	Control Mean	Control SD	Control Total	Weight	Std. Mean Difference IV, Random, 95% CI
**13.1.1 single TCMs**								
Congshan Li et al., 2021	-9.26	8.42	30	-2.64	8.67	30	4.7%	-0.76 [-1.29, -0.24]
Hongxia Li et al., 2020	-17.19	5.38	47	-12.71	5.31	49	5.1%	-0.83 [-1.25, -0.41]
Huifen Yang et al, 2016	-12.67	5.63	69	-0.1	6.49	69	5.2%	-2.06 [-2.47, -1.64]
Junyuan Cai et al., 2021	-20.34	9.31	30	-8.56	9.28	30	4.5%	-1.25 [-1.81, -0.69]
Ming Feng et al,2021	-22.27	3.46	45	0.79	3.63	45		Not estimable
Weikang Zhu,2020	-16.37	2.59	40	-9.61	5.12	40	4.7%	-1.65 [-2.16, -1.14]
Xiaoling Wang, 2018	-17.46	2.27	30	-13.54	2.42	30	4.4%	-1.65 [-2.24, -1.06]
Yemei Li et al, 2022	-12.45	5.07	25	-8.95	4.67	25	4.5%	-0.71 [-1.28, -0.13]
Yining Song et al, 2014	-19.15	6.27	60	1.9	3.922	60		Not estimable
**Subtotal (95% CI)**			**271**			**273**	**33.1%**	**-1.28 [-1.70, -0.86]**
Heterogeneity: Tau² = 0.25; Chi² = 28.93, df = 6 (P < 0.0001); I² = 79%								
Test for overall effect: Z = 5.98 (P < 0.00001)								
**13.1.2 TCMs with ER antagonists**								
Chen Nie, 2018	-6.7	7.91	20	4.5	7.69	20	3.9%	-1.41 [-2.11, -0.71]
Chen Sun, 2009	-24	8.76	30	-16.43	7.13	30	4.6%	-0.94 [-1.47, -0.40]
Chundi Gao, 2019	-20.13	6.36	24	1.71	4.86	24		Not estimable
Lan Luo et al, 2019	-4.5	14.9	30	1.34	7.41	30	4.7%	-0.49 [-1.00, 0.02]
Yang Fu, 2016	-28.96	7.53	50	-7.3	7.65	50		Not estimable
**Subtotal (95% CI)**			**80**			**80**	**13.3%**	**-0.90 [-1.40, -0.41]**
Heterogeneity: Tau² = 0.10; Chi² = 4.42, df = 2 (P = 0.11); I² = 55%								
Test for overall effect: Z = 3.57 (P = 0.0004)								
**13.1.3 TCMs with AI**								
Guilan Tan, 2014;	-7.96	5.38	28	-2.96	5.76	28		Not estimable
Han Xiao et al, 2019	-25.35	9.26	25	-11.74	9.27	25	4.2%	-1.45 [-2.07, -0.82]
Jingru Song, 2019	-14.04	4.9	27	-4.75	5.02	28	4.2%	-1.85 [-2.48, -1.21]
Kaili Xu, 2019	-20.95	5.32	50	-0.51	4.21	53		Not estimable
Limin Zhu,2020	-21.21	9.25	61	-0.78	9.76	60	5.0%	-2.14 [-2.58, -1.69]
Liujing Ou, 2018	-16.36	2.27	50	-11.27	2.42	50	4.8%	-2.15 [-2.65, -1.66]
Zhipei Han et al., 2021	-17.03	8.1	40	-1.64	8.59	40	4.7%	-1.83 [-2.35, -1.30]
**Subtotal (95% CI)**			**203**			**203**	**22.9%**	**-1.94 [-2.18, -1.69]**
Heterogeneity: Tau² = 0.00; Chi² = 4.08, df = 4 (P = 0.40); I² = 2%								
Test for overall effect: Z = 15.76 (P < 0.00001)								
**13.1.4 TCMs with endocrine therapy**								
Hui Zhang,2018	-25	6.14	30	-17.4	6.97	30	4.6%	-1.14 [-1.69, -0.59]
Junwen Pei et al, 2019	-15.7	4.65	30	-9.94	4.82	30	4.5%	-1.20 [-1.75, -0.65]
Lina Sheng, 2014	-27.04	6.13	23	-13.5	8.83	20	3.8%	-1.77 [-2.49, -1.05]
Rongfei Jiang, 2021	-4.21	2.51	29	-0.28	1.79	29	4.3%	-1.78 [-2.39, -1.16]
Ruiqing Sui, 2019	-6.94	9.46	29	1.96	11.04	30	4.6%	-0.85 [-1.39, -0.32]
Zhihui Tao et al, 2020	-10.83	2.23	30	-6.56	2.81	30	4.4%	-1.66 [-2.25, -1.07]
Ziyi Yan, 2021	-7.96	5.38	28	-2.96	5.76	28	4.6%	-0.88 [-1.44, -0.33]
**Subtotal (95% CI)**			**199**			**197**	**30.8%**	**-1.29 [-1.59, -1.00]**
Heterogeneity: Tau² = 0.07; Chi² = 10.68, df = 6 (P = 0.10); I² = 44%								
Test for overall effect: Z = 8.64 (P < 0.00001)								
**Total (95% CI)**			**753**			**753**	**100.0%**	**-1.38 [-1.61, -1.16]**
Heterogeneity: Tau² = 0.21; Chi² = 80.31, df = 21 (P < 0.00001); I² = 74%								
Test for overall effect: Z = 11.96 (P < 0.00001)								
Test for subgroup differences: Chi² = 20.94, df = 3 (P = 0.0001), I² = 85.7%								

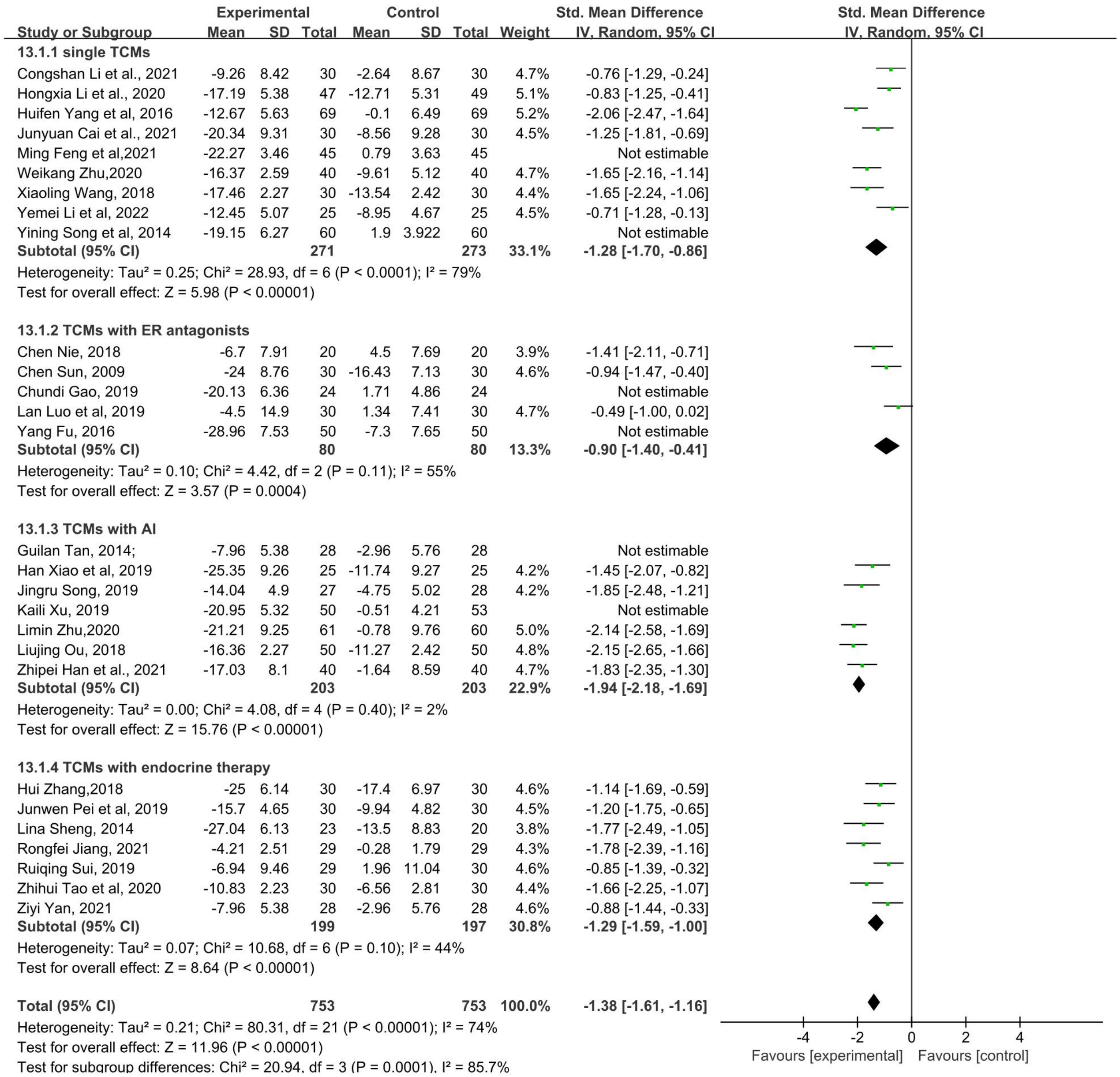

Figure S1. Sensitivity analysis showing the total score of KMI.

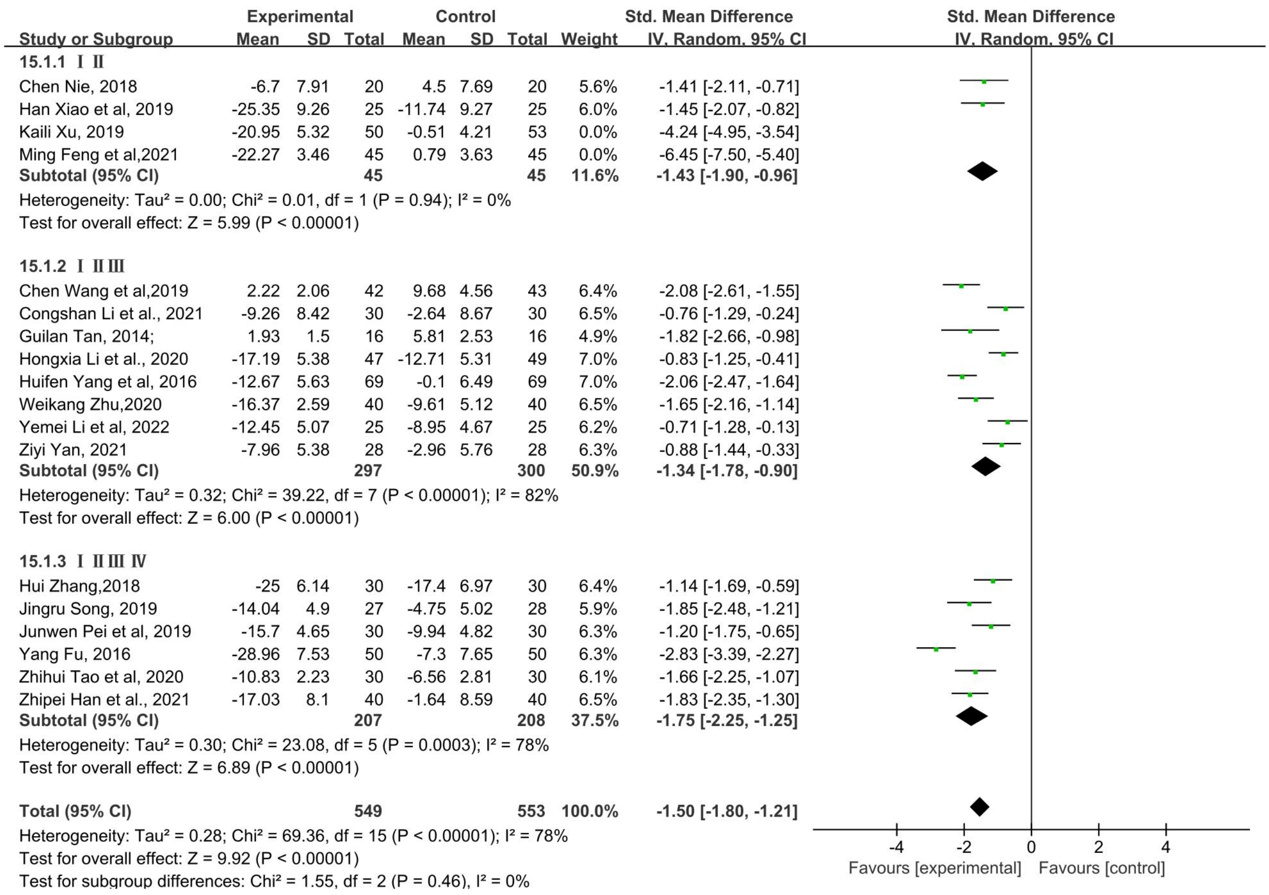

Figure S2. Sensitivity analysis showing the total KMI score based on tumour stage.

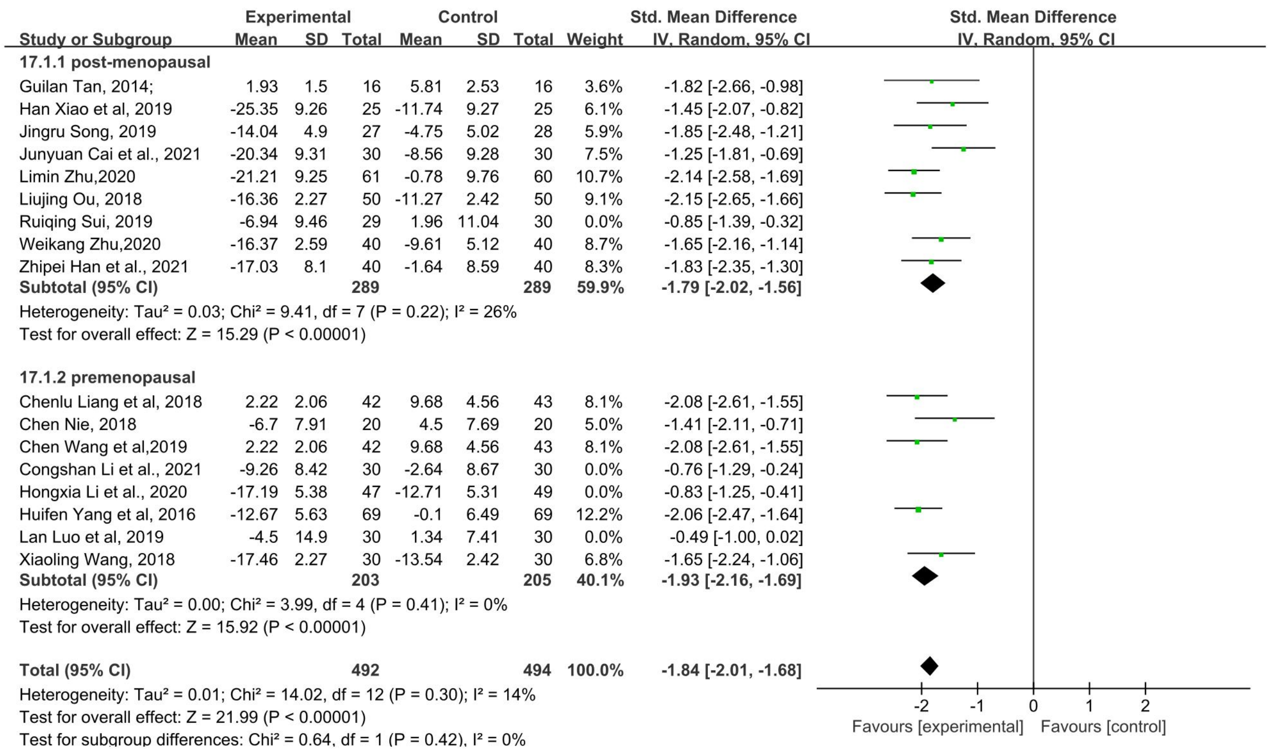

Figure S3. Sensitivity analysis showing the total KMI score based on the menstrual cycle.
