# Supplementary material for: Efficacy and safety of traditional Chinese medicine in the treatment of menopause-like syndrome for breast cancer survivors: a systematic review and meta-analysis
Source: BMC Cancer. 2024 Jan 8;24:42. doi: 10.1186/s12885-023-11789-z (PMC10773128; doi:10.1186/s12885-023-11789-z)
Supplement: Supplementary file 5 — Additional file 5. Quality of evidence assessment. [file 12885_2023_11789_MOESM5_ESM.pdf]

Table S2. Quality of evidence assessment

Participants (studies) Follow-up	Risk of bias	Inconsistency	Indirectness	Imprecision	Publication bias	Overall certainty of evidence	Study event rates (%)		Relative effect (95% CI)	Anticipated absolute effects	
							With Treatment of BC	With TCM		Risk with Treatment of BC	Risk difference with TCM
**Total score of KMI**											
2023 (28 RCTs)	serious [a]	not serious	not serious	not serious	none	⊕⊕⊕◯ Moderate	1010	1013	-	-	S MD **1.84 SD lower** (2.21 lower to 1.46 lower)
**Hot flashes and night sweats in KMI**											
387 (6 RCTs)	serious [a]	serious [b]	not serious	serious [c]	none	⊕◯◯◯ Very low	194	193	-	-	S MD **0.68 SD lower** (1.1 lower to 0.27 lower)
**Paresthesia in KMI**											
379 (6 RCTs)	serious [a]	not serious	not serious	serious [c]	none	⊕⊕◯◯ Low	190	189	-		MD **0.48 lower** (0.74 lower to 0.21 lower)
**Osteoarthralgia in KMI**											
420 (7 RCTs)	serious [a,d]	serious [b]	not serious	not serious	none	⊕⊕◯◯ Low	210	210	-	-	S MD **0.41 SD lower** (0.6 lower to 0.21 lower)
**Anxiety in KMI**											
432 (7 RCTs)	serious [a]	not serious	not serious	not serious	none	⊕⊕⊕◯ Moderate	216	216	-		MD **0.85 lower** (1.13 lower to 0.58 lower)
**Insomnia in KMI**											
479 (8 RCTs)	serious [a,d]	serious [b]	not serious	not serious	none	⊕⊕◯◯ Low	240	239	-	-	S MD **0.61 SD lower** (0.8 lower to 0.43 lower)
**ORR**											
871	not serious	serious [b]	not serious	not serious	none	⊕⊕⊕◯	250/439 (56.9%)	368/432 (85.2%)	not es timable	569 per 1,000	

(13 RCTs)						Moderate					
**E_2**											
1484<br><br>(21 RCTs)	serious [a,d]	very serious [b]	not serious	not serious	none	⊕◯◯◯<br><br>Very low	724	760	-	-	S MD **0.02 SD higher** (0.24 lower to 0.28 higher)
**FSH**											
1404<br><br>(20 RCTs)	serious [d]	serious [b]	not serious	not serious	none	⊕⊕◯◯<br><br>Low	685	719	-		MD **0.09 SD lower** (0.84 lower to 0.66 higher)
**LH**											
1081<br><br>(15 RCTs)	serious [d]	serious [b]	not serious	not serious	none	⊕⊕◯◯<br><br>Low	525	556	-		MD **0.99 lower**<br><br>(1.38 lower to 0.6 lower)
**AEs**											
1186<br><br>(6 RCTs)	not serious	serious [b]	not serious	not serious	none	⊕⊕⊕◯<br><br>Moderate	67/598 (11.2%)	45/588 (7.7%)	not es timable	112 per 1,000	

CI: confidence interval; **MD:** mean difference; **RR:** risk ratio; **SMD:** standardised mean difference

Explanations

a. Most information is from studies at unclear risk of bias.

b. Clinical heterogeneity exists owing to the different treatment plan.

c. Small sample size.

d. Potential limitations are likely to lower confidence in the estimate of effect.
